# ALDH 2 conferred neuroprotection on cerebral ischemic injury by alleviating mitochondria-related apoptosis through JNK/caspase-3 signing pathway

**DOI:** 10.7150/ijbs.38962

**Published:** 2020-02-17

**Authors:** Pingping Xia, Fan Zhang, Yajing Yuan, Cheng Chen, Yan Huang, Longyan Li, E Wang, Qulian Guo, Zhi Ye

**Affiliations:** 1 Department of Anesthesiology, Xiangya Hospital of Central South University, Changsha, 410078, Hunan Province, China.; 2 National Clinical Research Center for Geriatric Disorders, Central South University, Changsha, 410008, Hunan, P. R. China.; 3 Department of Anesthesiology, Tianjin Medical University Cancer Institute and Hospital, National Clinical Research Center of Cancer, Tianjin 300060, China.

**Keywords:** Stroke, OGD/R, ALDH2, mitochondria, apoptosis, JNK

## Abstract

Past studies have indicated that the dysregulation of Aldehyde dehydrogenase 2 (ALDH2) is related to the pathogenesis of acute stroke. However, the underlying mechanisms of ALDH2-mediated acute stroke are still not well understood. Thus, our study was designed to explore the influence of ALDH2 in acute stroke and determine whether its related mechanisms are involved in regulating mitochondria-associated apoptosis modulating JNK/caspase-3 pathway. *In vitro* analysis on the gain and loss of ALDH2 and JNK function were performed to explore its influence on OGD/R injury and relevant signaling pathways. Our findings suggested that ALDH2 expression was significantly down-regulated in rats suffering from acute stroke and also in primary cortical cultured neurons and PC12 cells upon OGD/R stimulation. ALDH2 overexpression markedly decreased infarct size and improved neurological outcomes. Furthermore, ALDH2 overexpression significantly suppressed stroke-induced mitochondria-associated apoptosis and inhibited p-JNK activation and p-JNK/caspase-3 complex formation. Similarly, in *in vitro* OGD/R models, ALDH2 reintroduction not only promoted cellular viability and moderated LDH release, but also inhibited mitochondria-related apoptosis. Moreover, JNK inhibition relieved OGD/R-induced cellular injury and apoptosis while JNK activation aggravated them. Furthermore, ALDH2 overexpression and JNK inhibition significantly reduced caspase-3 activation and transcription which was triggered by OGD/R damage. Caspase-3 activation and transcription also re-elevated during activation of JNK in ALDH2-reintroduced cells. Finally, ChIP assay revealed that p-JNK was bound to caspase-3 promoter. Collectively, ALDH2 overexpression led to a significant reduction in mitochondria-related apoptosis *via* JNK-mediated caspase-3 activation and transcription in both *in vitro* and *in vivo* cerebral ischemia models.

## Introduction

Stroke is the leading cause of death in Mainland China 1. Ischemic stroke is an extremely mortal cerebrovascular disease which has a high recurrence rate and results in disability. Stroke patients often cause a great deal of financial and psychological stress to their family. Despite continued advance in therapeutic techniques, a clear understanding of the pathophysiology and its mechanisms is still warranted.

In the event of an acute ischemic stroke, the neurons trigger a cascade of pathological events due to oxygen and energy supply loss and subsequently resulted in irreversible neuronal damage and impaired brain tissues. Emerging evidence has indicated that mitochondria play a pivotal role in the apoptosis in acute stroke attack, a process regulated by the apoptosis gene 2-4. Most of the apoptosis signaling pathways are directed to the mitochondria by altering its membrane potential and opening the permeability transition pore, which lead to leakage of substances from the mitochondria to the cytoplasm and the apoptosis of mitochondria and neurons. Mitochondria can thus be dubbed as the master switch of apoptosis 5, 6. Hence, therapeutic techniques aiming towards mitochondria-mediated apoptosis have become a novel strategy for prevention and treatment of acute stroke injury.

Aldehyde dehydrogenase 2 (ALDH2), located in the mitochondrial matrix, is encoded by a nuclear gene 7. It is most efficient in catalyzing high toxic acetaldehyde to low toxic carboxylic acid. ALDH2 is also associated with metabolizing other biogenic aldehydes in the brain, such as 4-hydroxynonenal (4-HNE) or malondialdehyde (MDA) 8, 9. Moreover, ALDH2 is widely expressed in both glial cells and neurons in the temporal and frontal cortex, hippocampus and the mid-brain, 10-12, suggesting a crucial function in protecting the neurons. Previous studies have demonstrated that cerebral ischemic reperfusion injury decreased ALDH2 expression, which was associated with higher contents of 4-HNE and MDA. They also suggested that enhanced ALDH2 activity protected the brain from ischemic injury by cleaning 4-HNE and MDA 13, 14.

Excessive levels of reactive oxygen species (ROS) related to stroke-impaired mitochondria initiate apoptosis signaling pathways and are accompanied by an imbalance between pro-oxidants and antioxidants 4, 15, 16. c-Jun N-terminal kinase (JNK), a subtype of the mitogen-activated protein kinase (MAPK) superfamily, participates in numerous neuronal oxidative stress injury models and also has a critical role in mediating neuron apoptosis 17, 18, 19. More and more evidence suggested that ROS served as a potential inducer of JNK 20, 21 and activation of JNK triggered neuronal apoptosis under brain ischemic conditions 18, 22, 23. Additionally, both extrinsic and intrinsic pathways of apoptosis eventually merge in caspase cascade, with caspase-3 activation considered as the final step. Previous experimental evidence demonstrated that JNK signaling pathway was an upstream mediator of ischemic damage induced apoptosis through the control of caspase-3 activation 24, 25. Moreover, ALDH2 is crucial in the regulation of ROS generation 26, 27. ALDH2 deficiency leads to elevation of ROS levels 28. Although JNK is activated while ALDH2 is inhibited after suffering from stroke, the direct relationship between activated JNK and ALDH2 has not been examined. Thus, there are enough reasons to speculate that enhancing ALDH2 activity disturbs the balance of cellular redox initiated by excessive ROS and ultimately leads to suppressed JNK activation and neuronal apoptosis.

In summary, we focused on the relationship between brain ischemia and ALDH2 expression, and aimed to seek out the underlying molecular mechanism of ALDH2-induced neuroprotection. Therefore, we adopted an *in vivo* rat focal cerebral ischemic model together with an *in vitro* neuronal cell oxygen-glucose deprivation/re-oxygenation (OGD/R) model. It was hypothesized that the possible regulatory mechanism of knockdown and overexpression of ALDH2 was involved in mediating mitochondria-related apoptosis *via* JNK-caspase-3 signaling pathway.

## Materials and Methods

### Specimen origin

Upon gaining approval from the ethics committee of Xiang Ya Hospital, Central South University, male adult Sprague-Dawley rats weighing 250-300 g, were purchased from Cavens Co.,Ltd. (#2007-04, Changsha, Hunan, China) and utilized in accordance with the guidelines of the Care and Use of Laboratory animals. Water and food were supplied and the temperature was set at 24±1°C with alternating 12-hour light and darkness.

### Establishment of a MCAO animal model and experimental design

Anesthesia was performed with pentobarbital sodium (30 mg/kg, intraperitoneal injection), and 100% oxygen was supplied through masks. Middle cerebral artery occlusion (MCAO) was induced according to the method described in our previous studies 29, 30. The middle cerebral artery was occluded for 90 min before 24 h of reperfusion. Laser Doppler imaging was applied to confirm ischemia and reperfusion. Rectal temperature was controlled at 37°C while 0.25% bupivacaine hydrochloride was given at the incision sites for postoperative analgesia.

Then, the subjects were randomly divided into four groups (*n*= 6-8 in each group): (1) Sham group: rats that underwent surgical procedures but had no ischemia; (2) MCAO group: rats with 90 min of ischemia followed by 24 h reperfusion; (3) ALDH2-OE group: rats receiving intra-cerebroventricular injection of lentivirus-ALDH2 expressing vectors one week before MCAO. (4) ALDH2-NC group: rats receiving intra-cerebroventricular injection of lentivirus expressing negative control.

### Infarct volume and neurological deficit score assessment

A neurological score was evaluated 24 h after reperfusion using a 5-point rating scale: point 0 = no deficit, point 1 = failure to extend the left forepaw, point 2 = decreased grip strength of the left forepaw, point 3 = circling to left by pulling the tail, point 4 = spontaneous circling.

The animals (n=6, per group) were euthanatized after neurological deficit evaluation. Infarct volumes were assessed by staining in 1% 2,3,5-triphenyltetrazolium chloride (TTC; Sigma-Aldrich). To rule out the impact of post-stroke edema to ischemic size, we employed a modified equation: corrected infarct volume = volume of the contralateral hemisphere - (volume of the ipsilateral hemisphere - detected infarct volume) 31. The infarct size was represented as the proportion of the total brain volume and was measured blindly.

### Assessment of morphology by TUNEL staining

Brains were extracted and fixed with 4% formaldehyde for 24 h at 4°C before embedding in paraffin for sectioning. Peri-infarct tissue was sectioned at 4 μm and underwent staining with an TUNEL Apoptosis Assay Kit (provided by Beyotime, PRC). The number of TUNEL positive neurons was captured by using a light microscope (provided by Olympus Corporation, Japan) and quantified using Image Pro Plus 6.0 (offered by Media Cybernetics Inc., Bethesda, America).

The TUNEL-positive primary cortical neurons emitted green fluorescent light under One Step TUNEL Apoptosis Assay Kit (#C1086, Beyotime Biotechnology, Shanghai, China). However, the TUNEL-positive astrocytes emitted red fluorescent light under TUNEL FITC Apoptosis Detection Kit (#A111-01, Vazyme Biotech Co., Ltd. Nanjing, China). All the treated cells were observed under a fluorescence microscope (Olympus, X51) and analyzed by using Image-Pro Plus 6.0

### Measurement of 4-HNE and MDA

Briefly, ischemic penumbral cortex and cells were procured from frozen PBS and processed using an ultrasonic disruption system at 5000 × g for twenty minutes. The supernatant was obtained with 4-HNE and MDA content measured by a commercial ELISA kit (provided by R&D, Minneapolis, America).

### Isolation of mitochondria

The mitochondrial fraction of the harvested brain tissues and cells was obtained according to the previous method 32. The cytosolic and mitochondrial parts of the ischemic penumbra and cells were processed with a Mitochondria Isolation Kit for Cultured Cells in accordance with the manual (provided by ThermoFisher Scientific, America). The lack of cytoplasmic calnexin in Western blotting analysis (data concealed) showed efficient isolation of mitochondria.

### Measurement of mitochondrial membrane potential, mitochondrial permeability transition pore opening and mitochondrial ROS formation

After 24 h of reperfusion, the ischemic penumbral cortex of experimented animals was collected. Then, alterations in the membrane potential of mitochondria were done with the fluorescent dye Rhodamine 123 (Rh123) 16. Mitochondrial permeability transition pores (MPTP) opening was evaluated using a kit provided by GENMED SCIENTIFICS INC. Finally, mitochondrial ROS formation was monitored using dichlorodihydrofluorescein diacetate (DCFH-DA) assay. All procedures adhered to the producers' manuals.

### Immunofluorescent staining

Sections were incubated with the primary antibody 1:200 ALDH2, 1:1000 NeuN and 1:100 GFAP (Abcam, Cambridge, MA) overnight at 4 °C. They were then incubated with 1:100 goat anti-mouse or goat anti-rabbit secondary antibodies (Zhongshan Goldbridge Biotechnology, Beijing, PRC) under 25°C for 60 min. Results were observed through a fluorescence microscope (IX81; Olympus).

### Western blot analysis and immunoprecipitation

The method was described previously 33. Briefly, cells were lysed with RIPA buffer with protease inhibitors for extraction of the proteins. BCA Protein Assay Kit (provided by Com Win Biotech Co., Ltd., Beijing, China) was then used to measure its concentration. Proteins were divided by (w/v) SDS-PAGE (12%) before being moved to a PVDF (polyvinylidene fluoride) membrane (purchased from Millipore, Bedford, MA). It was put overnight under 4°C in TBST with the primary antibodies after processing in skimmed milk (5%) for 60 min under room temperature: 1:1000 ALDH2 (Abcam), 1:1000 Cyt C (Abcam), 1:1000 phosphorylated JNK (Abcam), 1:1000 JNK (Abcam), 1:1000 active caspase-3 and -9 (Santa Cruz), 1:500 Bcl-2 and Bax (Cell Signaling), and 1:2000 VDAC (Abcam) or 1:2000 GAPDH (Zhongshan Golden Bridge Biotechnology). Following washing in TBST for three rounds, the membrane was incubated with the secondary antibody for 60 min and processed with TBST for another three rounds (five minutes each). The signals were picked up using the enhanced chemiluminescence detection kit (provided by ECL, Pierce Biotechnology Inc.). The density was measured using Quantity One software. VDAC and GAPDH were applied for control of intrinsic quality. Ratios of the target protein against the internal reference yielded relative expression levels.

The specimens were then put together with antibody-bound protein A/G-Agarose beads (offered by Santa Cruz Biotechnology) throughout the night at 4 °C. Following washing, the beads were re-suspended in pH 3.0 100 mM glycine for 10 seconds before mixing with a pre-titrated pH 9.5 1.0 M Tris to reach neutral. Western blotting was used to evaluate the supernatants.

### Isolated and cultured the primary cortical neurons and astrocytes

The primary cortical neurons extracted from embryo cerebrums of Sprague-Dawley rats (16-18 days) were used in this research. The cell suspensions were seeded on six-well plates (concentration: 1.5×10^5^ cells per well) and neurobasal medium containing 2% B27 (Gibco, USA) was used for culture under 5% CO_2_ in a 37°C incubator, which was added with glutamine (2 mM) and penicillin/streptomycin (50 U/ml) 34.

Cerebral astrocytes were taken from neonatal Sprague-Dawley rats. Briefly, neonatal rats on postnatal day 1 (P1) were anaesthetized with 2% isoflurane and the cortex was dissected and digested with trypsin for 15 min at 37 °C. Cells were placed on dishes containing 0.0125% poly-L-lysine-coated culture in Neurobasal medium (Gibco, USA) with B27 (Gibco, USA) and were incubated in a humidified atmosphere with 5% CO_2_ at 37 °C. Half of the culture medium was renewed every 3 days. When the cell confluence reached 85-95%, the cells were shaken at 37 °C at 300rpm for 5 h. The adherent cells were treated with trypsin and passaged, and those at passage 3 were used for subsequent experiments 35.

### Cell culture and OGD/R treatments

Cells were cultured under 37 °C at a humidified atmosphere with 5% CO_2_ utilizing 5mM of DMEM containing basal glucose along with 10% FBS. An oxygen-glucose deprivation/re-oxygenation (OGD/R) plot was built based on the previous method 35, 36. In short, the cells were processed with glucose-free Earle's BSS (balanced salt solution) at 37 °C in an oxygen-less chamber of N_2_ (95%) and CO_2_ (5%) for 6-hour OGD treatment. They were then put into a normal neurobasal medium and recovered at thirty-seven degrees Celsius under a humidified environment of CO_2_ (5%). The control group underwent similar procedures, except for OGD exposure.

To overexpress ALDH2, pcDNA-ALDH2 plasmids were built by Shanghai GeneChem Co., Ltd. An empty pcDNA3.1 vector served as control. Cells were transfected with either pcDNA-ALDH2 or empty pcDNA3.1 utilizing Lipofectamine 2000 (offered by Invitrogen Life Technologies). Following 2 days of transfection, the medium was changed into fresh DMEM 24 h following transfection and the cells were processed for subsequent experiments. To knock down the ALDH2 expression, siRNA transfection was performed ahead of OGD/R induction with Lipofectamine 2000, in accordance with the producer's guidelines. Cells were treated with ALDH2-siRNA or a negative control that did not match the sequence of ALDH2 (ALDH2-Ctr) for 1 day to compare with DMEM containing 2% (v/v) FBS and then brought to OGD/R treatment.

Subsequently, cells were separated into 6 groups: (1) Control group (under normal condition): cells were not treated by OGD; (2) OGD group: cells were treated by OGD for 6 h and reoxygenation for 24 h; (3) OGD + ALDH2 overexpression (ALDH2+OE) group: Cells were treated with pcDNA-ALDH2 for 48 h, and then subsequently subjected to OGD for 6 h and reoxygenation for 24 h; (4) OGD + ALDH2-NC group: Cells were infected with empty pcDNA3.1 and followed the same experimental protocol as above. (5) OGD + ALDH2 knockdown group (ALDH2-KD): Cells were processed with ALDH2-siRNA 24 h before OGD, and then suffered from OGD for 6 h and reoxygenation for 24 h; (6) OGD+ALDH2-Ctr group: Cells were infected with negative control siRNA and followed the same experimental protocol as above. For pharmacological approaches, JNK specific inhibitor or activator: SP600125 (SP, 10 μM, purchased from Selleck Chemicals) and Anisomycin (Ani, 10 μM, purchased from Selleck Chemicals) and were added to PC12 cells 2 h before treatment respectively.

### Cell survival, LDH release and caspase-3 activity

Cell counting kit-8 (CCK-8) (from Dojindo, Japan), LDH cytotoxicity detection kit (provided by Roche, Basel, Switzerland) and Caspase-3 activity analysis (from Beyotime, China) were used as previously described 37.

### Acridine orange (AO)/ Propidium iodide (PI) staining

Acridine orange (AO)/ propidium iodide (PI) staining was reported before 37. PC12 cells were cultured into 24-well plates before being stained with PBS, 25 mg/ml ethidum bromide and 5 mg/ml acridine orange. All cells could be stained by acridine orange, while only cells with membrane pores allowed diffusion of ethidium. The apoptotic ratio was counted and shown as the ratio of PC12 cells with positive ethidium bromide staining.

### Real‑Time PCR

Total RNA was removed using the RNA isolation reagent TRIzol (Invitrogen,) and was reverse transcribed using a cDNA reverse transcription kit (Biosystems, Foster City, CA, USA) following manufacturer instructions. The following primer sequences were utilized for real-time PCR: caspase-3: 5'-AGGTGGGCATCTGGTAGCCA-3' and 5'-GATCAGTTATCGCGAATGCCA-3'; and GAPDH: 5'-ACGGCCA-GGTCATCACTATTG-3' and 5'-CCTGCTTGCTGATCCACATCT-3'. Real-time PCR assays were performed using 2 × SYBR Green PCR Master Mix (Biosystems, Warrington, UK) and a Bio-Rad CFX96 Real-Time System (Singapore) according to manufacturer instructions. There were three replicates for each sample. The experimental cycle threshold (CT) value was regularized to β-actin, which was quantified on the same plate. The fold differences in gene expression were calculated with the 2-ΔΔCt method 32.

### Chromatin immunoprecipitation assay

Chromatin immunoprecipitation was done as in previous studies 38. First, PC12 cells were washed and fixed in formaldehyde (1%) for 20 min before sonicated to shear chromatin. Antibodies against histone H3 antibodies against p-JNK were added to the cleared lysate with the binding taking place at 4°C overnight. Immuno-precipitated DNA was released from histones, processed with proteinase K, treated with phenol‐chloroform extraction and ethanol precipitation, and re-suspended in PCR‐grade water. PCRs were performed and the products were subjected to agarose gel electrophoresis, which were indicated by ethidium bromide.

### Statistics

Except for neurological deficit scores, data were portrayed as mean ± standard deviation (SD). SPSS 18.0 statistic software (provided by SPSS Inc., Chicago, America) was used for data processing and Student's t-test, one-way ANOVA and Bonferroni post hoc test were applied for the difference. Kruskal-Wallis test and Mann-Whitney U test with Bonferroni correction were used for the neurological deficit scores. P<0.05 indicated significant difference.

## Results

### The alternation of ALDH2 expression after acute stroke injury

**Figure 1:**
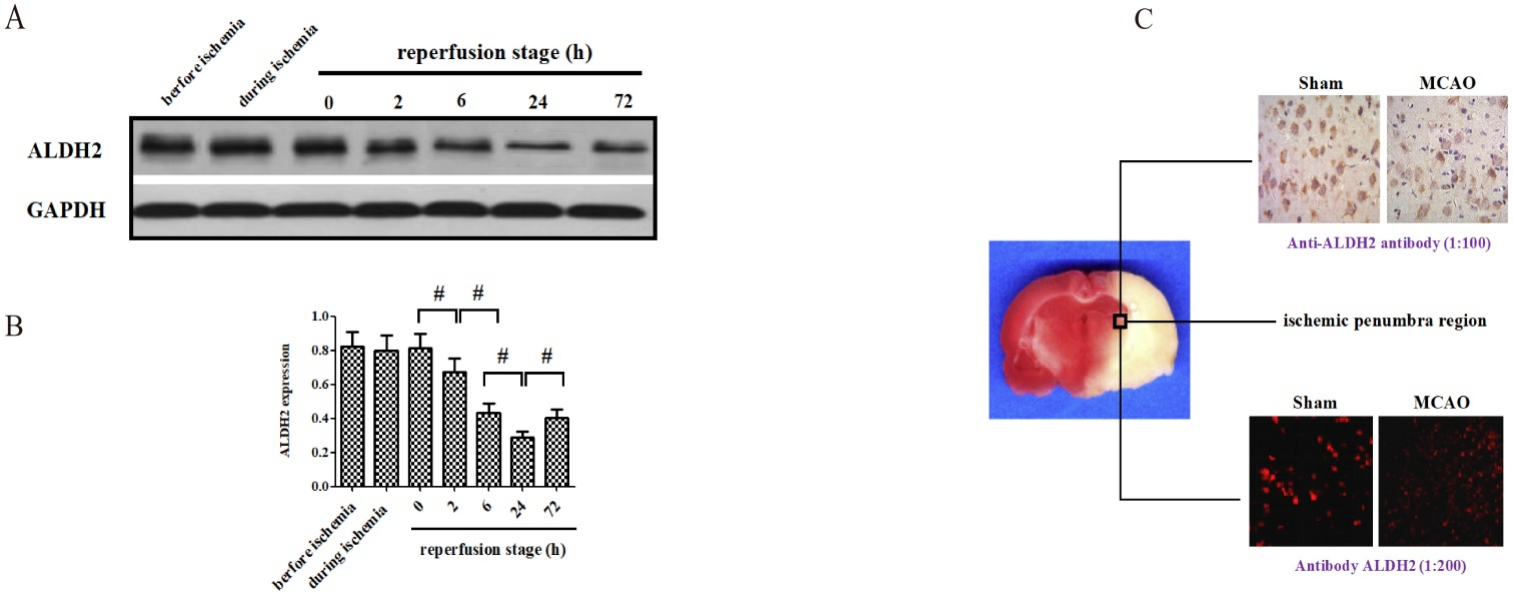
** Effects of cerebral ischemia-reperfusion injury on the expression of ALDH2. (A).** ALDH2 was significantly down-regulated at different time points after reperfusion (the lowest level at 24h after reperfusion). **(B).** Western blot analysis showed ALDH2 expression in the ischemic penumbra of animals treated with middle cerebral artery occlusion (MCAO). **(C).** Immunohistochemistry and immunofluorescence staining showed that ALDH2 was significantly decreased in MCAO group, in comparison with those in the sham group. Mean **±** SD was used to describe the data (n = 6-8 in each group). ^#^ indicated P < 0.05.

At the onset, changes of ALDH2 expression were detected before, during and after focal cerebral ischemic-reperfusion. Compared with no-infarcted period (before MCAO), the level of ALDH2 in the ischemic penumbra did not experience significant change during the ischemic period and the immediate aftermath of reperfusion. However, the expression of ALDH2 significantly decreased at various time points following reperfusion, suggesting the content of ALDH2 was down-regulated in the reperfusion stage rather than in the period of ischemia (**Fig. 1A and B**). Additionally, the level of ALDH2 was lowest at 24 h after reperfusion and rose again at 72 h after reperfusion. Hence, reperfusion for 24 h was used for subsequent experiments. With further help from immunohistochemistry and immunofluorescence staining, the ALDH2 protein expression in the ischemic penumbra of MCAO group was found to be markedly lower than the sham group's non-infarcted zone. (**Fig. 1C**).

### ALDH2 overexpression contributed to the neuroprotection against acute stroke attack

**Figure 2:**
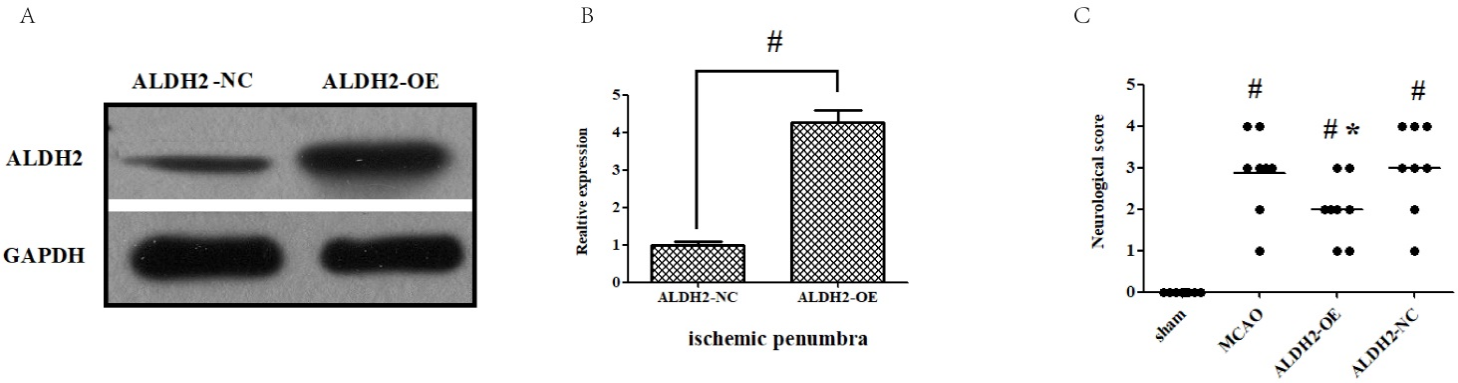

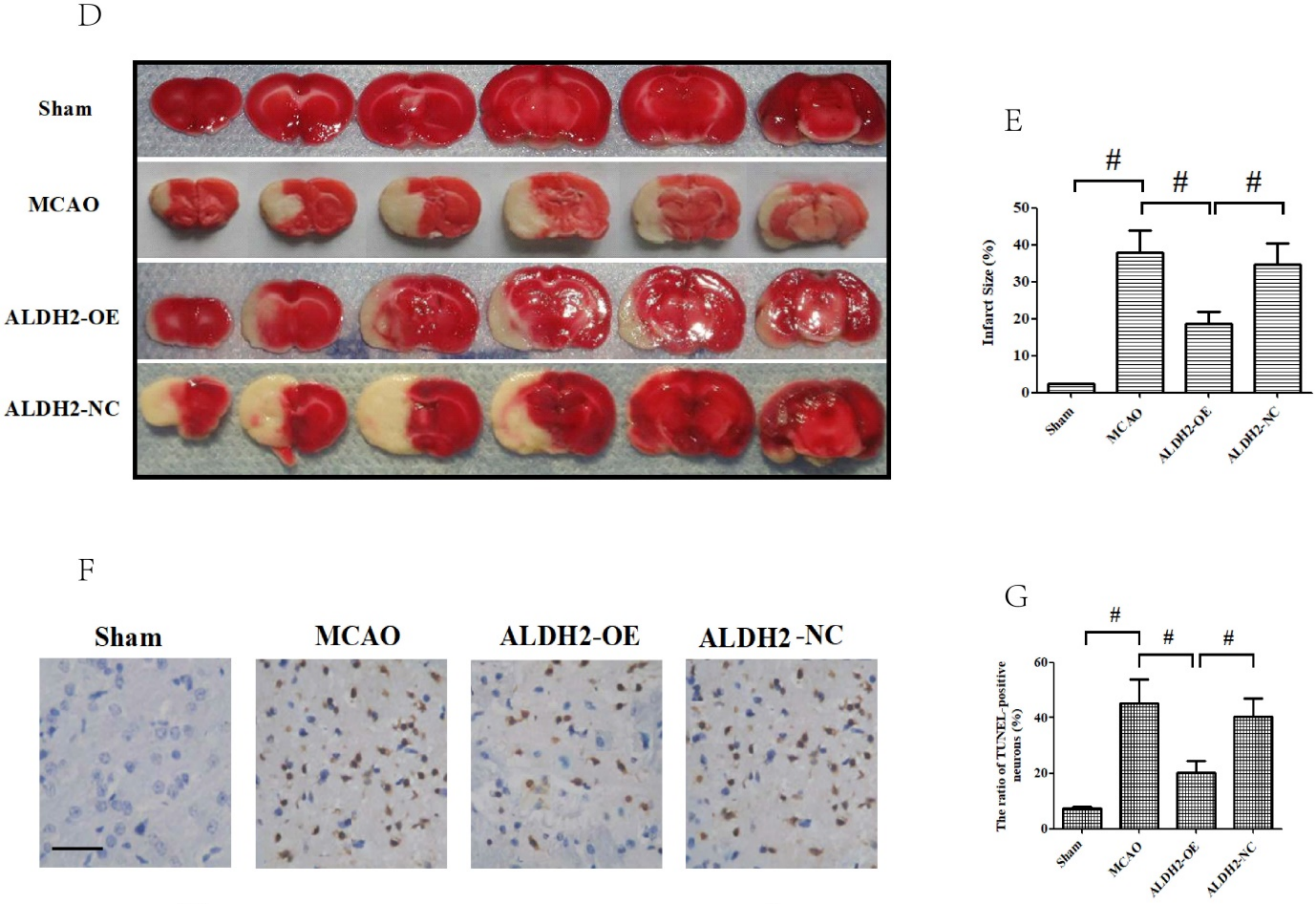

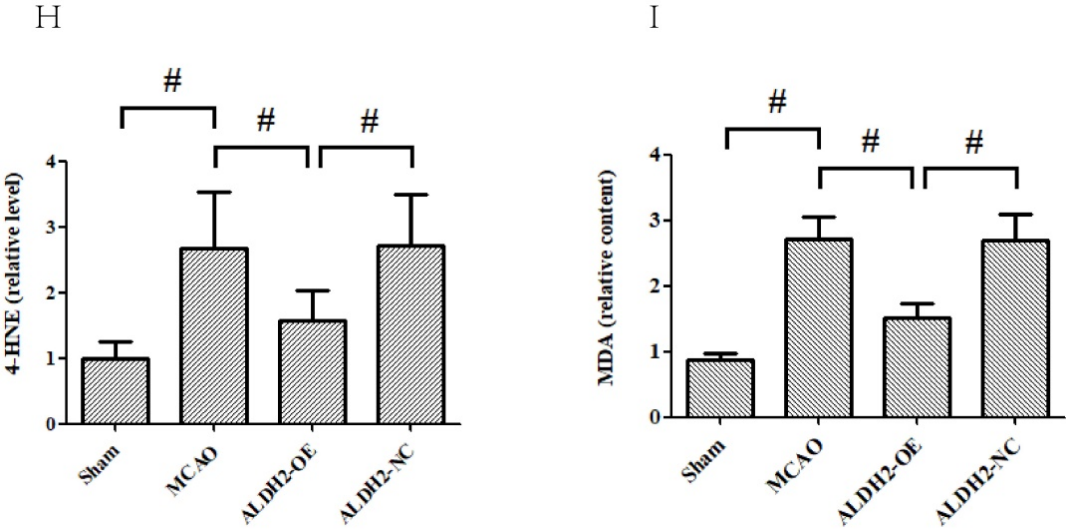
** Overexpression of ALDH2 confers a robust neuroprotective effect against acute stroke. (A, B)** Overexpression of ALDH2 expression was achieved by lentivirus transfection and confirmed by Western blot analysis. **C.** Neurological deficit scores of each group. ^*,^ *P* **<** *0.05, compared to* MCAO group;^ #^*P* **<** *0.05, compared to* sham group. **D.** Selected images of TTC-stained brain slices. The white areas are defined as the infarct regions. **E.** Statistical analysis of infarct volume percentages.** F.** Selected photographs of *in situ* detection of apoptotic neurons by TUNEL staining; **G.** Percentage of TUNEL-positive nuclei in the ischemic penumbra. Scale bar, 100μm. **H. and I.** Quantitative analysis of relative 4-HNE and MDA content respectively. Sham, sham-operated; MCAO, focal cerebral ischemia-reperfusion injury induced by middle cerebral artery occlusion; ALDH2-OE, the MCAO model was established one week after intracerebroventricular injection of lentivirus-ALDH2 overexpressing vectors. ALDH2-NC, the MCAO model was set up one week after intracerebroventricular injection of lentivirus of negative control vectors. Mean **±** SD was used to describe the data (n = 6-8 in each group). ^#^ indicated P< 0.05.

Firstly, intra-cerebroventricular administration of lentivirus-ALDH2 expressing vectors significantly increased the ALDH2 expression in cortical penumbral area. Meanwhile, negative vectors had no impact, which indicated that ALDH2 overexpression rats were successfully bred (**Fig. 2A and B**). Neurological deficit scores (NDS) were used to evaluate the neurological outcome and all animals were found to have developed severe neuron-related deficits 24 h after reperfusion, except for the sham group. Compared with those in MCAO and ALDH2-NC group, rats in the ALDH2 group improved at 24 h after MCA occlusion. (**Fig.2C**).

Moreover, the infarct volume percentage among the entire ischemic hemisphere was determined by TTC staining. Animals in the ALDH2-OE group had significantly lower infarct volumes, compared to the ALDH2-NC group (**Fig. 2D and E**). Finally, the cellular damage under ALDH2 overexpression was checked by TUNEL assay. Almost none of the TUNEL-positive cells were seen among the sham group. Nevertheless, the ALDH2 overexpression rats had lower number of TUNEL-positive cells within the cortical penumbral zone than the animals in the MCAO and ALDH2-NC group (**Fig. 2F and G**). Therefore, these findings suggested that overexpression of ALDH2 notably alleviated the severity of pathological changes after acute stroke injury.

4-hydroxynonenal (4-HNE) and malondialdehyde (MDA), identified as reactive aldehydes, can result in tissue damage by degrading many biological macromolecules. Given that both 4-HNE and MDA are classic toxic aldehydes, their contents were measured. Compared with the sham group, both 4-HNE and MDA were significantly raised in the MCAO and ALDH2-NC group. However, ALDH2 overexpression reduced the content of 4-HNE and MDA, suggesting ALDH2 reintroduction effectively inhibited toxic aldehyde accumulation (**Fig. 2H and I**).

In addition, physiological variabilities including MAP, blood glucose levels, rectal temperature, HCT, blood pH, and blood PO_2_ or PCO_2_ were detected before MCAO, 60 min after MCAO, and 60 min after reperfusion. None of these parameters were different between the lentivirus-ALDH2 expressing vector and negative vehicle-treated rats (data concealed). Moreover, we did not observe any obvious alternation in the residual CBF except for the sham group during ischemia period (data were not shown).

### ALDH2 alleviated mitochondrial damages and mitochondria-associated apoptosis *via* downregulating p-JNK

**Figure 3:**
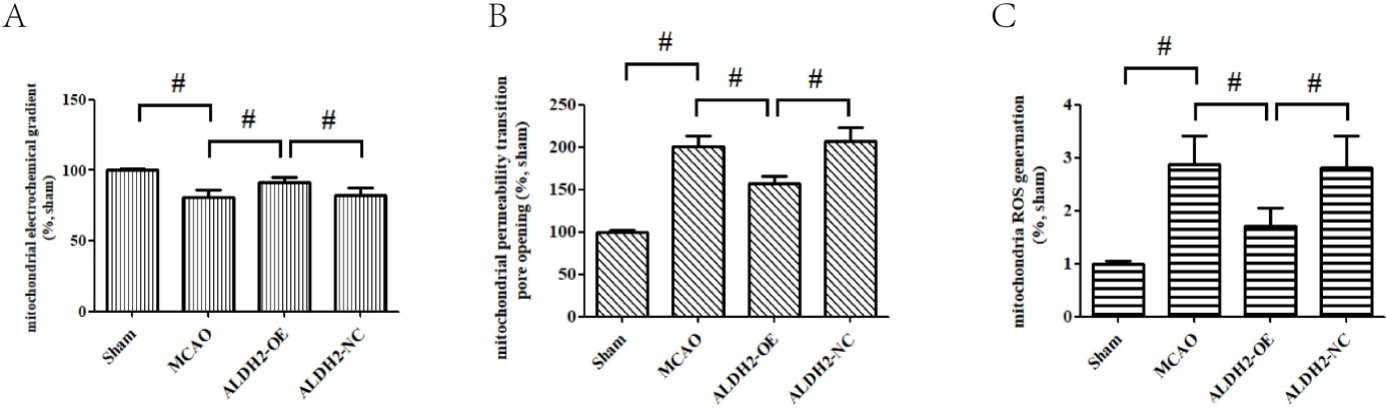

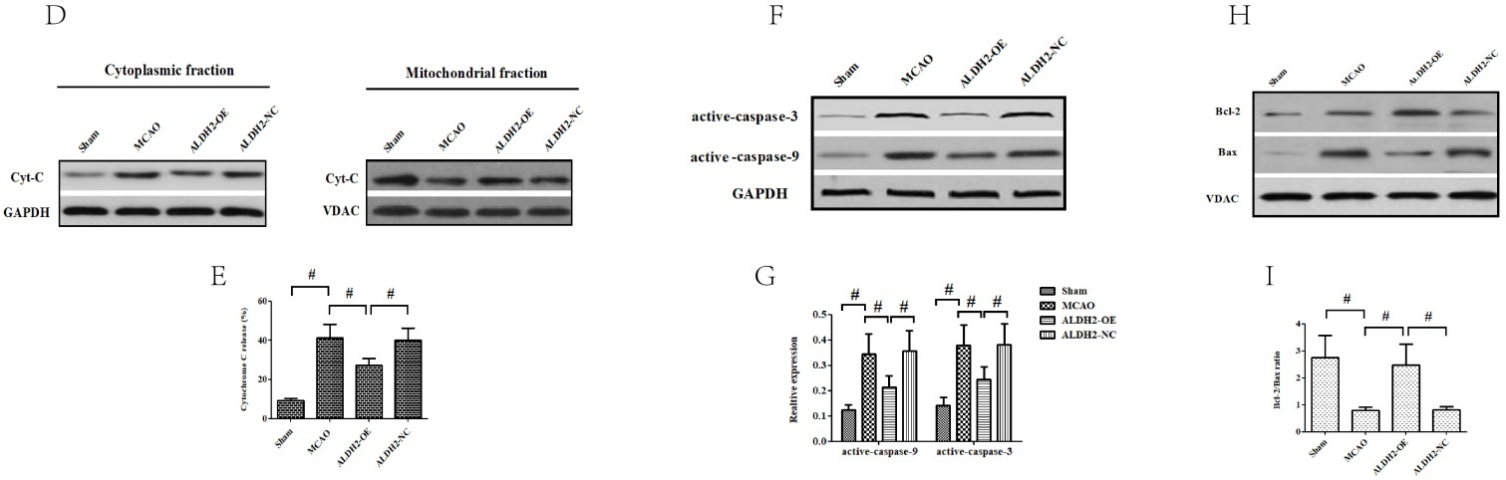

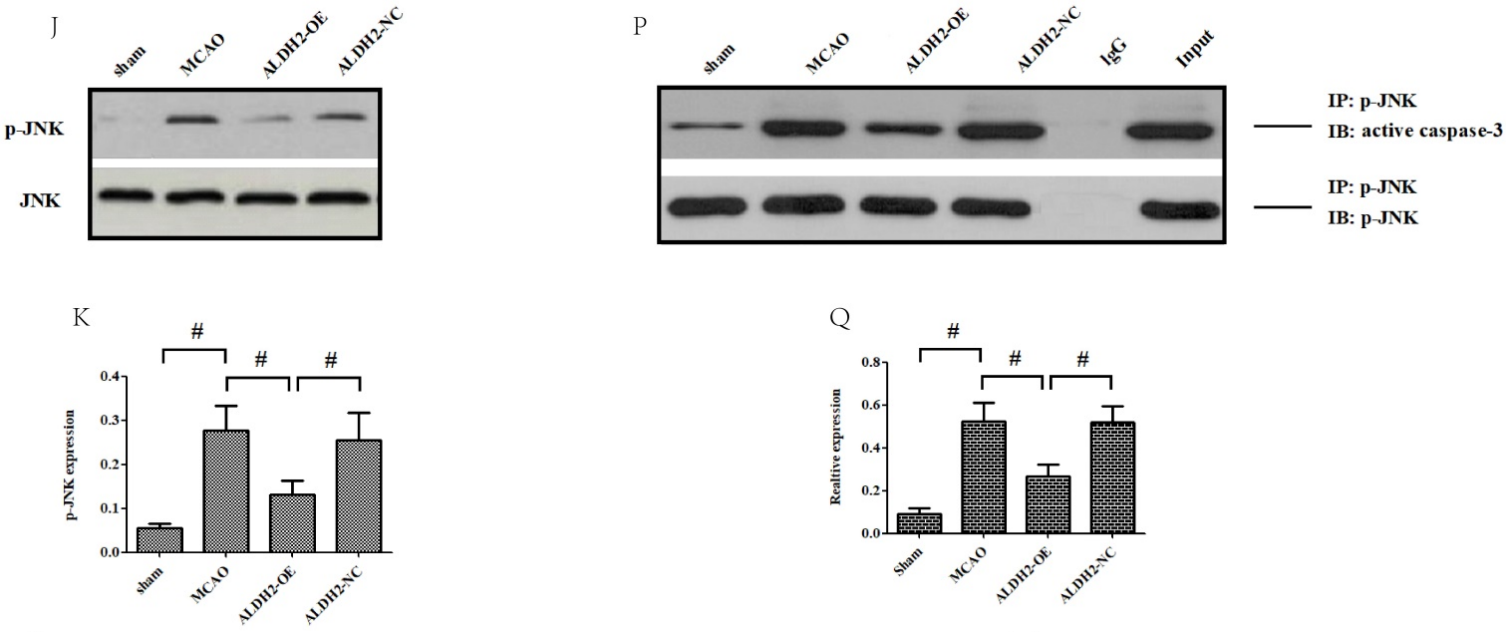

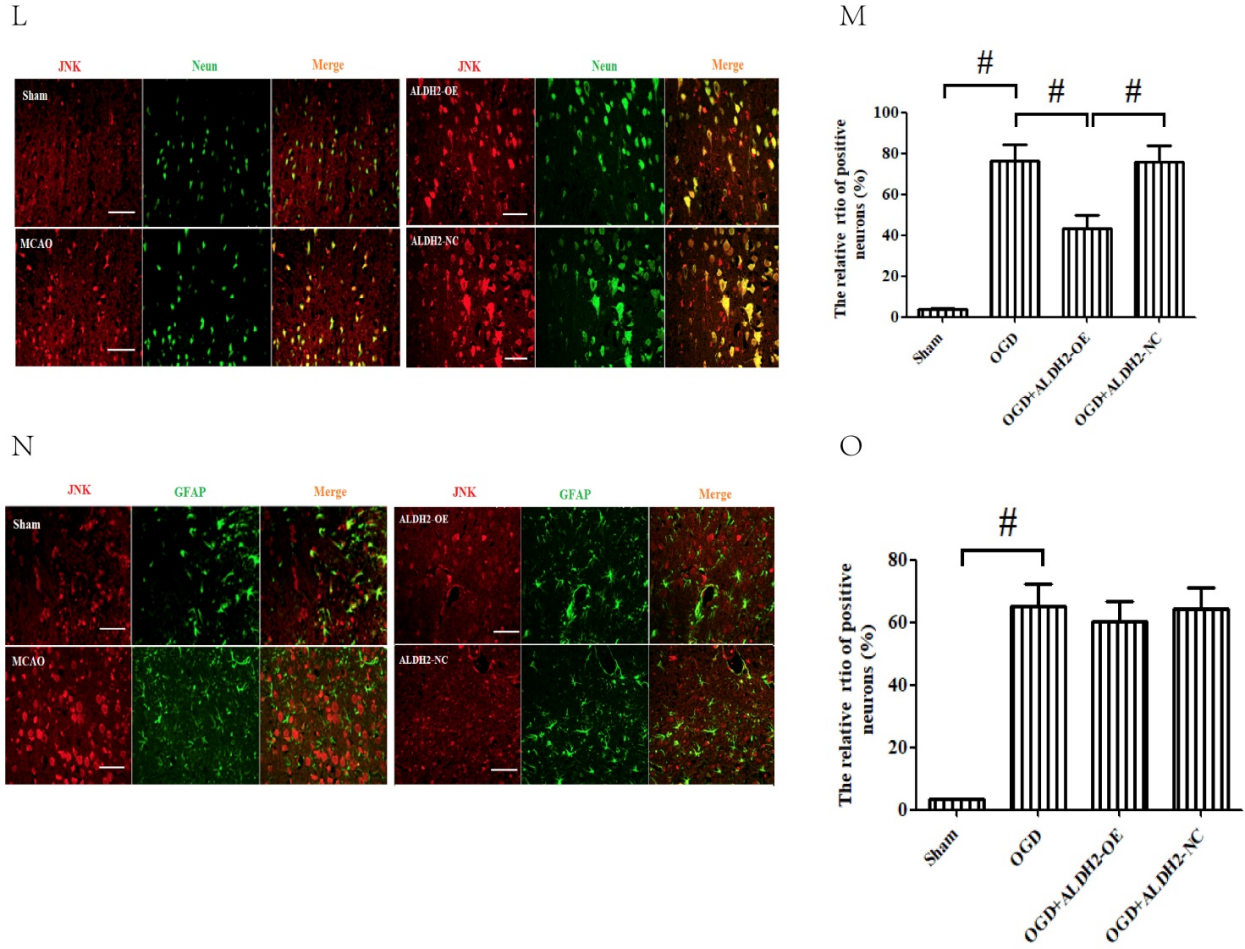
** ALDH2 overexpression alleviated MCAO-induced mitochondrial dysfunction and mitochondria-relative apoptosis. A.** ALDH2 overexpression preserved the stability of mitochondrial electrochemical gradient (ΔΨm); **B.** ALDH2 overexpression inhibited mitochondrial permeability transition pore (mPTP) opening; **C.** ALDH2 overexpression suppressed mitochondrial ROS generation; **D.** Release of Cyt-C from the mitochondria to cytoplasm; **E.** The relative release rate of Cyt-C from the mitochondria to cytoplasm; **F.** Selected plots of active caspase-9 and -3 in different groups; **G.** Relative optical density of active caspase-9 and -3. **H.** Selected plots of Bcl-2 and Bax expression in mitochondria in different groups;** I.** Relative ratio density of Bcl-2/Bax in mitochondria. **J.** Phosphorylated JNK (p-JNK) and JNK expression in different groups; **K.** Relative optical density of p-JNK and JNK; **L -O.** Coronal sections of the ischemic penumbra of MCAO rats with or without treatment of lentivirus overexpressing ALDH2. These were immunostained with an anti‐p-JNK (red) and an anti-Neun or an anti‐glial fibrillary acidic protein (GFAP, green), Magnification, 400×, Scale bar: 100 μm. **P and Q.** Immunoprecipitation and immunoblotting assays showing p-JNK and caspase-3 binding after transient MCAO**.** Mean **±** SD was used to describe the data (n = 6-8 in each group). ^#^ indicated P < 0.05.

Cerebral ischemia reperfusion injury is associated with a decline in mitochondrial fitness. Thus, to obtain more concrete proof of the neuro-protective role of ALDH2, our study focused on mitochondrial fitness. Firstly, acute stroke impaired mitochondrial electrochemical gradient (ΔΨm), as shown by the decreasing ΔΨm content. However, ALDH2 overexpression maintained the stability of ΔΨm (**Fig. 3A**). In addition, focal cerebral ischemia-reperfusion injury resulted in increased mitochondrial permeability transition pore (mPTP) opening, which was usually at normal levels after ALDH2 overexpression (**Fig. 3B**). As depicted in** Fig. 3C**, focal ischemia-reperfusion injury promoted mitochondrial ROS production (mROS) in ischemic penumbra of the MCAO and ALDH2-NC group. ALDH2 overexpression also inhibited excessive ROS generation in the mitochondria. Furthermore, western blot analysis demonstrated that acute stroke attack led to a significant increase in the release of mitochondrial pro-apoptotic factor Cyt-C into the cytoplasm. Similarly, ALDH2 overexpression markedly suppressed the Cyt-C leakage (**Fig. 3D and E**). In addition, other pro-apoptotic proteins associated with the mitochondrial-related apoptosis, such as active-caspase-9 and -3 and mitochondrial Bax, were elevated in rats suffering from MCA occlusion but were lowered in rats transfected with LV-ALDH2. On the other hand, the anti-apoptotic protein Bcl-2 was significantly decreased after acute stroke attack before being reversed after ALDH2 overexpression (**Fig. 3F-I**).

To further explore the mechanism by which ALDH2 mediated the acute stroke injury, we subsequently evaluated the phosphorylated JNK (p-JNK) expression and found that it was stroke that mainly increased p-JNK expression. Overexpression of ALDH2, on the other hand, significantly relieved the activation of p-JNK (**Fig. 3J and K**). Using immunofluorescence analysis, we unexpectedly observed that p-JNK positive cells, which were reduced by ALDH2 overexpression, were located mostly at neurons (Neun-staining) instead of astrocytes (GAFP-staining) (**Fig.3L-O**). Additionally, p-JNK and caspase-3 complex formation's response to acute stroke was also evaluated although their interaction was significantly reduced by ALDH2 overexpression (**Fig. 3P and Q**).

### Gain and loss of ALDH2 function altered cellular viability upon **oxygen-glucose deprivation/re-oxygenation (OGD/R)** stimulation

**Figure 4:**
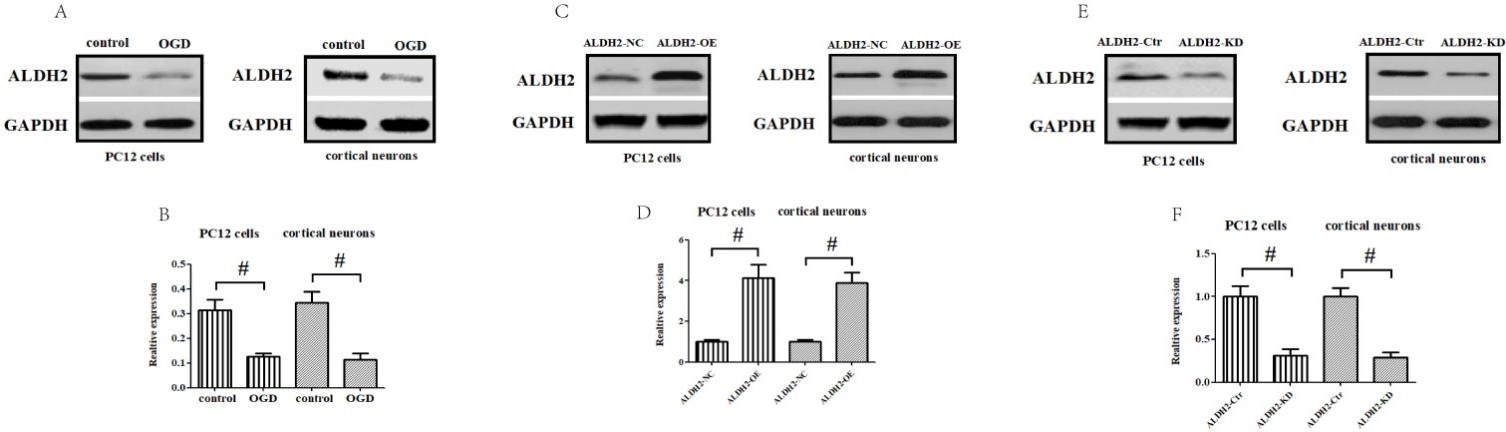

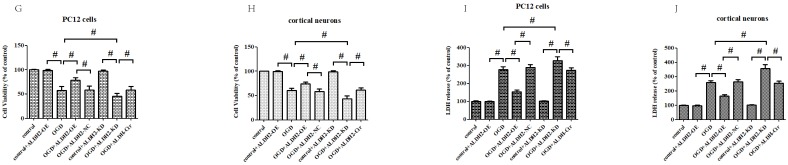

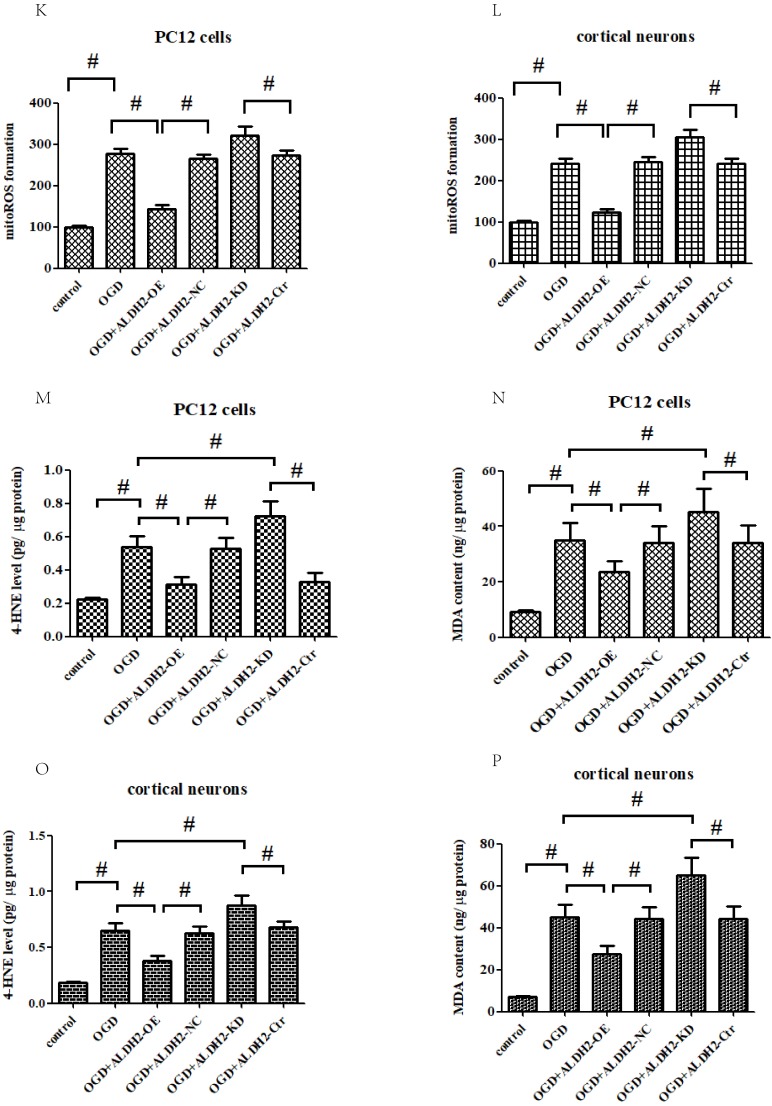
** Effects of reintroduction or inhibition of ALDH2 on the cellular viability of PC12 cells exposed to OGD injury. A and B.** OGD injury reduced ALDH2 protein levels as shown in the Western blot image. GAPDH was used as a loading control. Quantitative data of ALDH2 levels are shown. **C and D.** Overexpression of ALDH2 was achieved by plasmid transfection and verified by Western blot. **E and F.** ALDH2 siRNA reduces ALDH2 levels in the primary cortical neurons and PC12 cell lines. **G -J.** The effect of overexpression or knockdown of ALDH2 on cell survival and LDH leakage of the primary cortical neurons and PC12 cells under OGD conditions. K and LALDH2 overexpression or inhibition alleviated or aggravated mitochondria-derived ROS production. M-P. The level of MDA and 4-HNE in the primary cortical neurons and PC12 cells detected by ELISA kits.

To obtain more solid evidence, we mimicked the cerebral ischemic injury in the primary cortical neurons and PC12 cells using the OGD/R model, respectively. Being consistent with the previous results, the ALDH2 expression was significantly reduced against control after 6-h of OGD and 24 h of reoxygenation both in the primary cortical neurons and PC12 cells (**Fig. 4A and B**). Then, a function assay of ALDH2 was performed by using pcDNA3.1 and siRNA. The transfection efficiency was verified by western blots (**Fig. 4C-F**). In the meantime, cellular viability and LDH release following transfection were detected. Reintroduction or knockdown of ALDH2 had no effect on the viability of the cells and LDH release under normal conditions. However, overexpression of ALDH2 promoted OGD/R-induced cellular survival and reduced LDH leakage. ALDH2 inhibition led to an opposite effect and deteriorated OGD/R-induced damage both in the primary cortical neurons and PC12 cells (**Fig. 4G-J**).

Apart from cell viability, we also detected mitochondrial ROS (mROS) formation. Our findings demonstrated that OGD/R injury significantly increased mROS generation when compared to control conditions. ALDH2 overexpression markedly reduced the production of OGD/R-induced excessive mROS, while inhibition of ALDH2 aggravated mROS production both in the primary cortical neurons and PC12 cells (**Fig. 4K and L**). Furthermore, OGD/R-induced oxidative stress of the primary cortical neurons and PC12 cells can be measured by both the intracellular 4-HNE and MDA level. ALDH2 reintroduction or knockdown alleviated or exacerbated the aggregation of 4-HNE and MDA (**Fig. 4M -P**).

**Figure 5:**
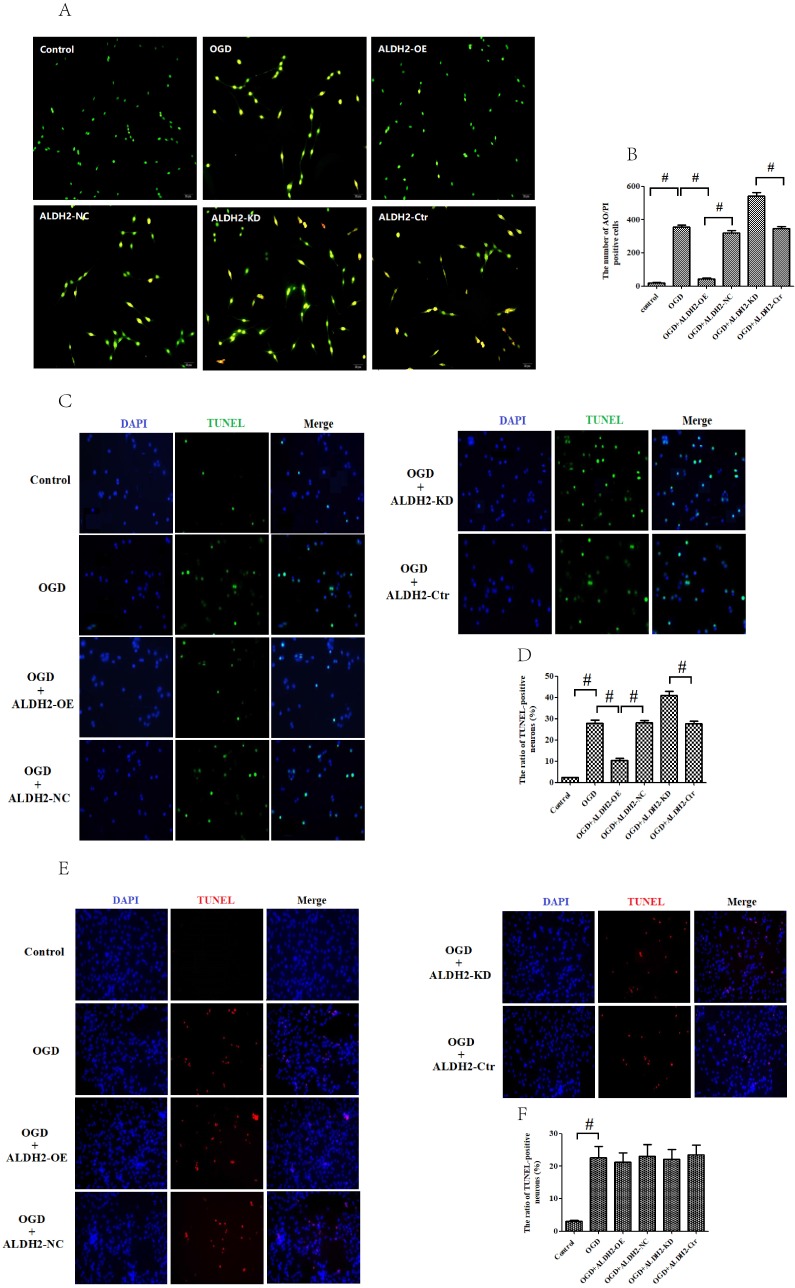

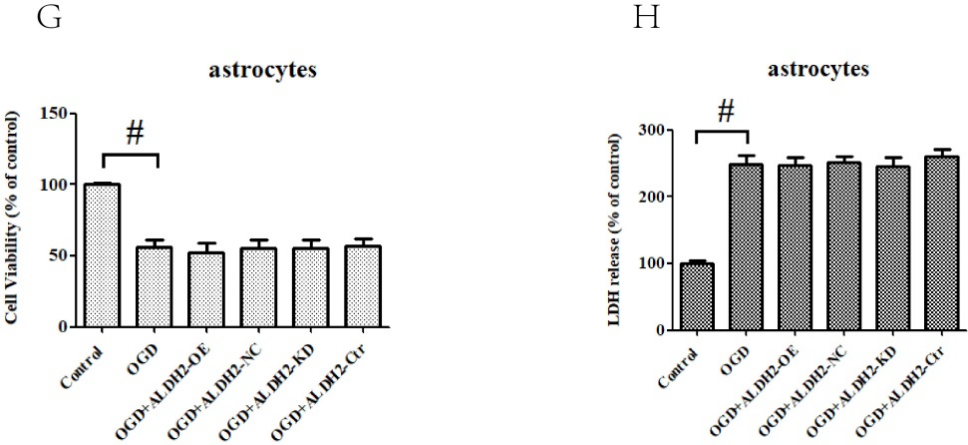
** ALDH2 modulated apoptosis in OGDR-treated neurons. A and B.** The effect of overexpression or knockdown of ALDH2 on apoptosis using AO/PI staining. **C-F.** TUNEL staining for the primary cortical neurons and astrocytes apoptosis. Representative images are shown (magnification, 200×). Scale bar: 100 μm. Representative fluorescence images and quantitative analysis of 3 independent experiments. **G and H.** Neither ALDH2 overexpression nor inhibition had any impact on cellular survival of cultured astrocytes. Mean **±** SD was used to describe the data (n = 6-8 in each group). ^#^ indicated *P < 0.05*.

Moreover, we detected the apoptosis of PC12 cells by using acridine orange (AO)/ propidium iodide (PI) staining. As evidenced in **Fig. 5A and B**, the green fluorescence indicated the normal cells, and orange fluorescence revealed the apoptotic cells. Similarly, reintroduction of ALDH2 decreased AO/PI positive staining cells when compared with OGD/R-treated group while ALDH2 knockdown increased AO/PI positive staining cells. It was suggested that the reintroduction of ALDH2 prevented OGD/R injury by inhibiting apoptosis, while inhibition of ALDH2 had opposite effects.

In order to obtain more direct evidence, TUNEL staining was used to assess OGD/R-induced neuronal apoptosis in the primary cortical neurons and astrocytes. OGD/R damage caused marked increase of neuronal apoptosis in both the primary cortical neurons and astrocytes. In contrast with data from MCAO model, ALDH2 reintroduction significantly decreased the numbers of TUNEL-positive cortical neurons and improved cellular viability, whereas ALDH2 knockdown had an opposite influence (**Fig. 5C-F**). However, neither overexpression nor inhibition of ALDH2 modulated the cultured astrocytes death, as shown by cellular viability and LDH release (**Fig. 5G and H).**

### ALDH2 regulated mitochondrial-related apoptosis *via* JNK

**Figure 6:**
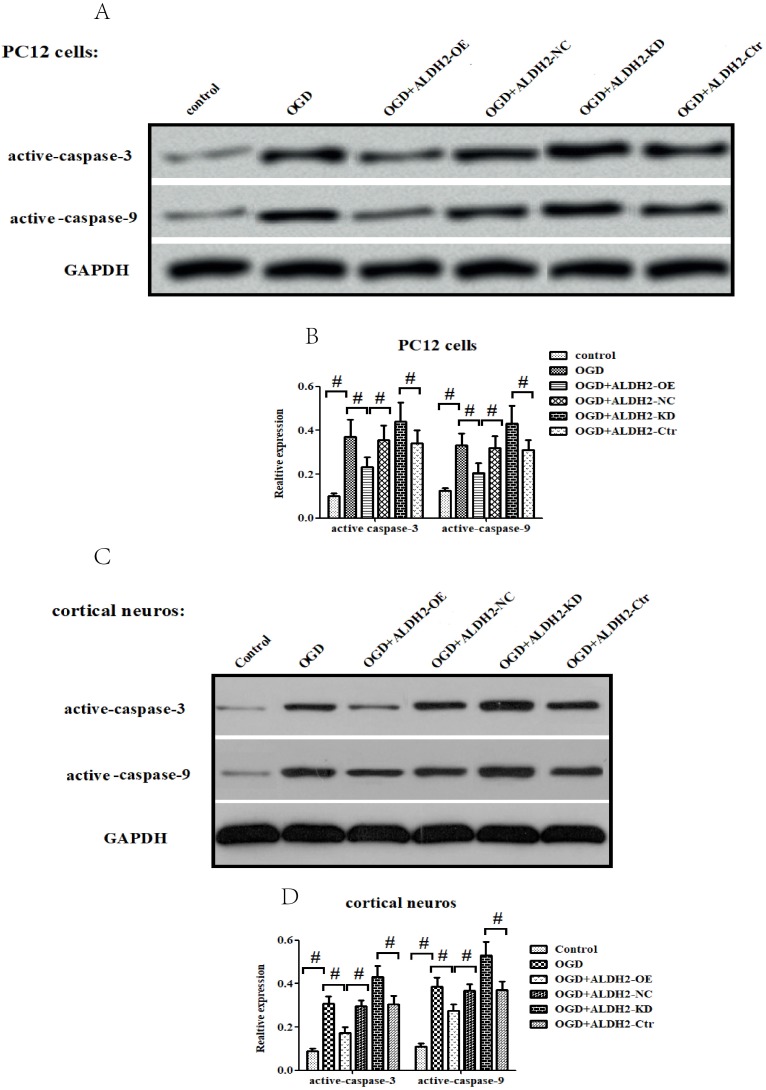

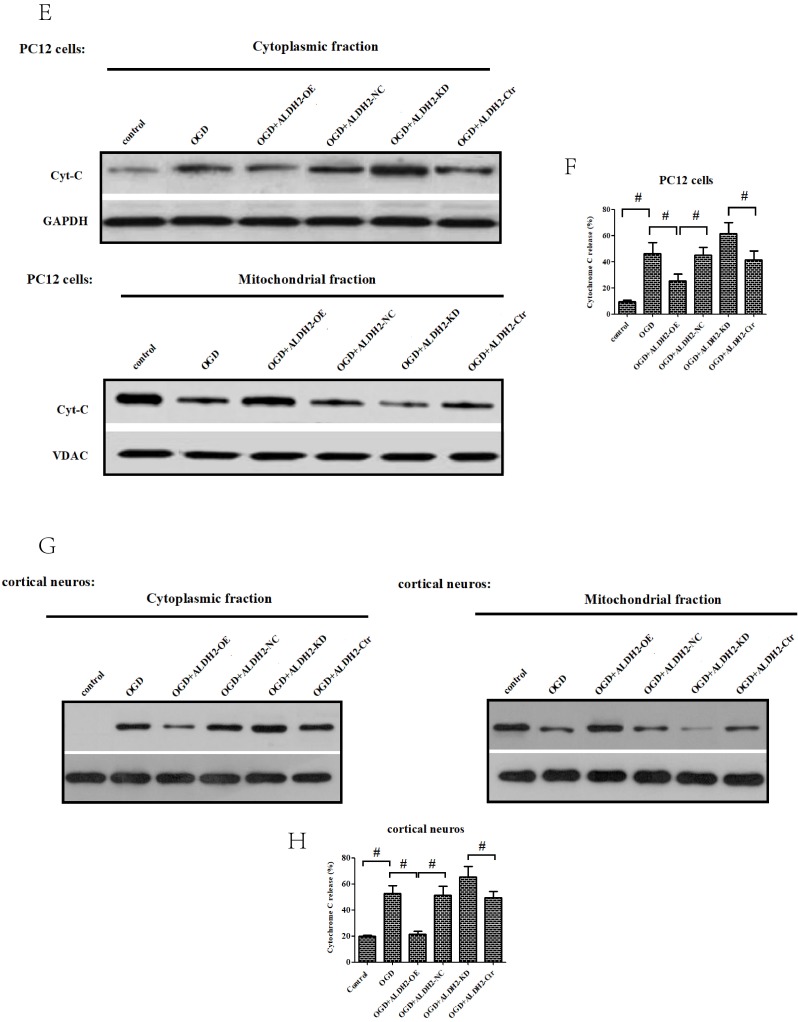

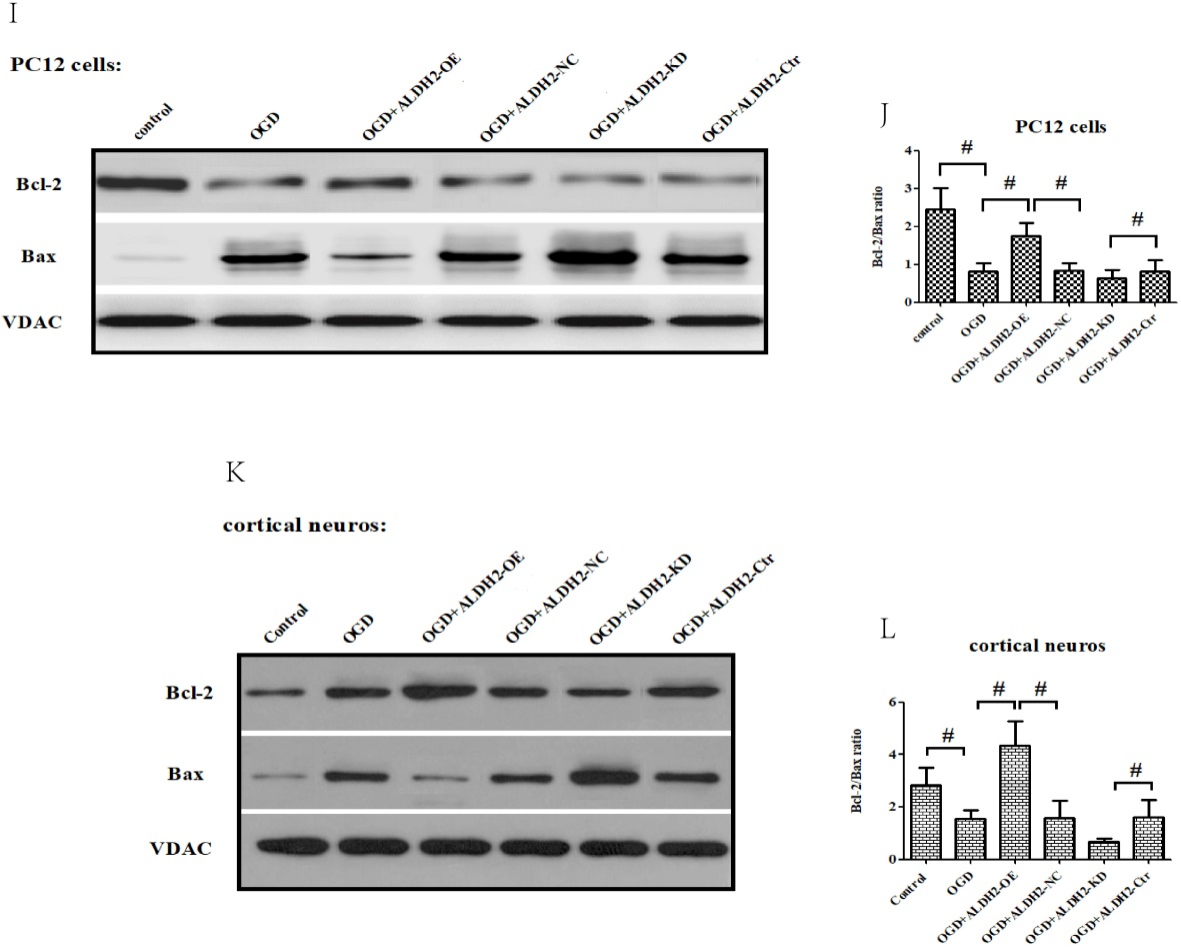
** ALDH2 was involved in mitochondria-dependent apoptosis. A-D.** The effect of overexpression or knockdown of ALDH2 on caspase-3 and -9 activation in the primary cortical neurons and PC12 cells exposed to OGD damage. **E-H.** Effects of overexpression or knockdown of ALDH2 on Cyt C release in the primary cortical neurons and PC12 cells exposed to OGD damage. **I-L.** Effects of overexpression or knockdown of ALDH2 on the Bcl-2/Bax ratio in the primary cortical neurons and PC12 cells exposed to OGD damage. Mean **±** SD was used to describe the data. ^#^ indicated *P < 0.05*.

In accordance with* in vivo* models, the exposure to OGD/R attack in primary cortical neurons and PC12 cells resulted in a significantly increased expression of activated caspase-9 and -3 (**Fig. 6A -D**) as well as Cyt C release from mitochondria to cytoplasm (**Fig. 6E-H**). OGD damage also up-regulated Bax but down-regulated Bcl-2 in mitochondria. These data suggested that OGD/R injury caused a marked mitochondria-associated apoptosis in the primary cortical neurons and PC12 cells. However, the reintroduction of ALDH2 alleviated OGD/R-induced apoptosis and the knockdown of ALDH2 promoted the apoptosis induced by OGD/R (**Fig. 6I-L**).

**Figure 7:**
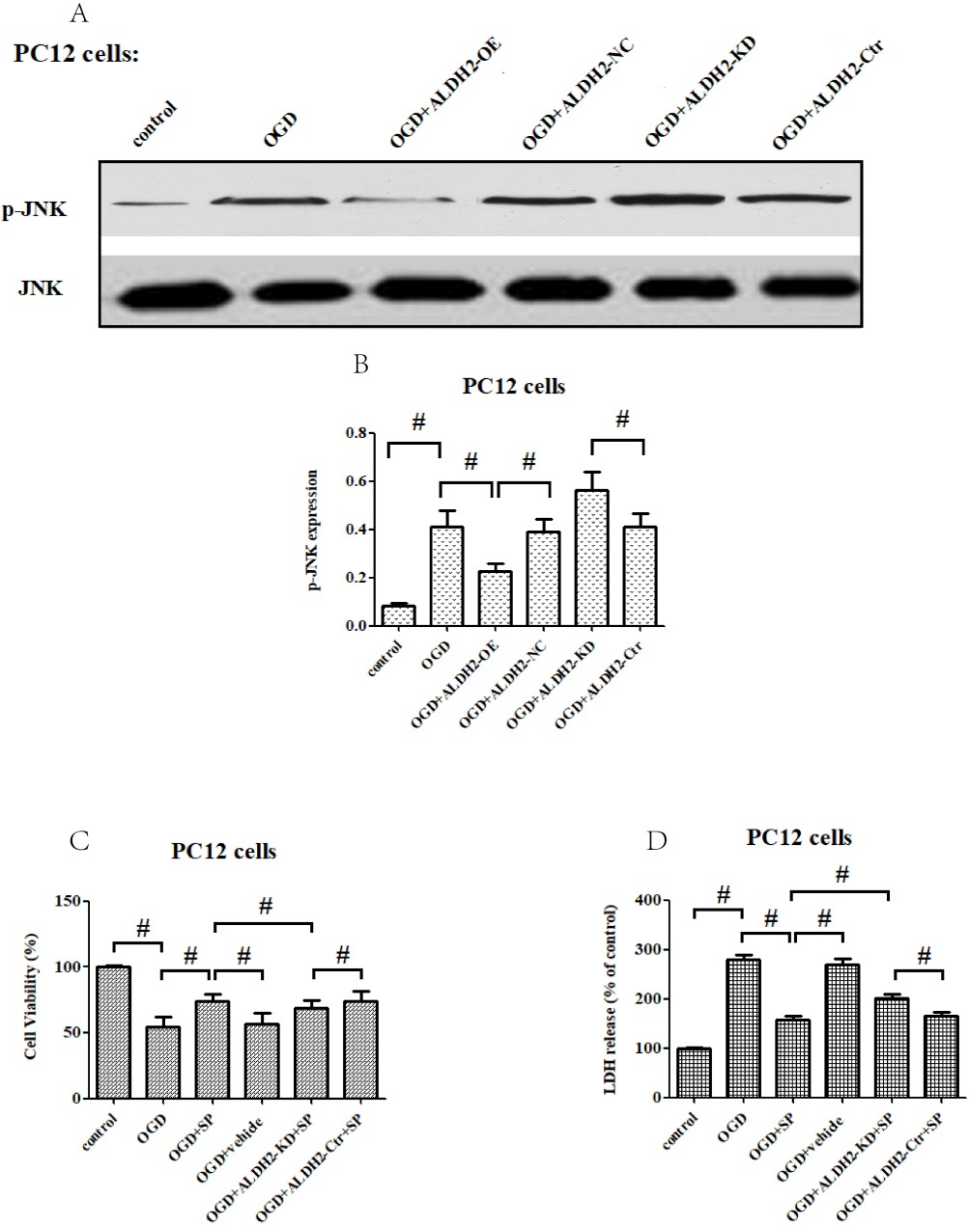

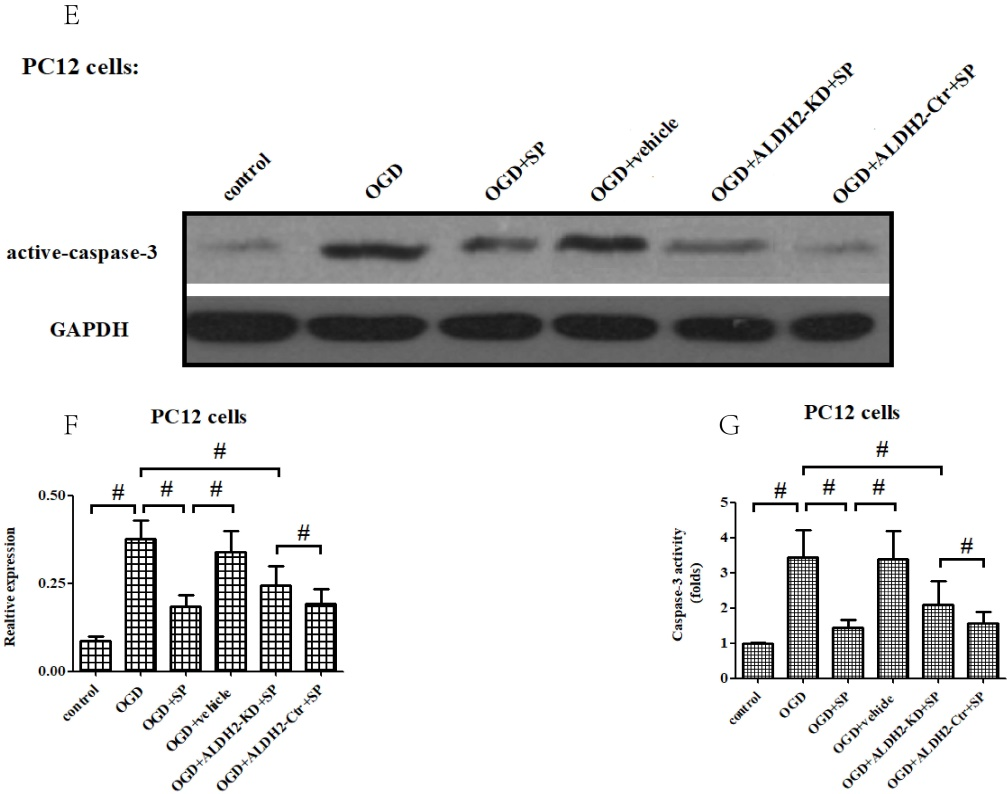

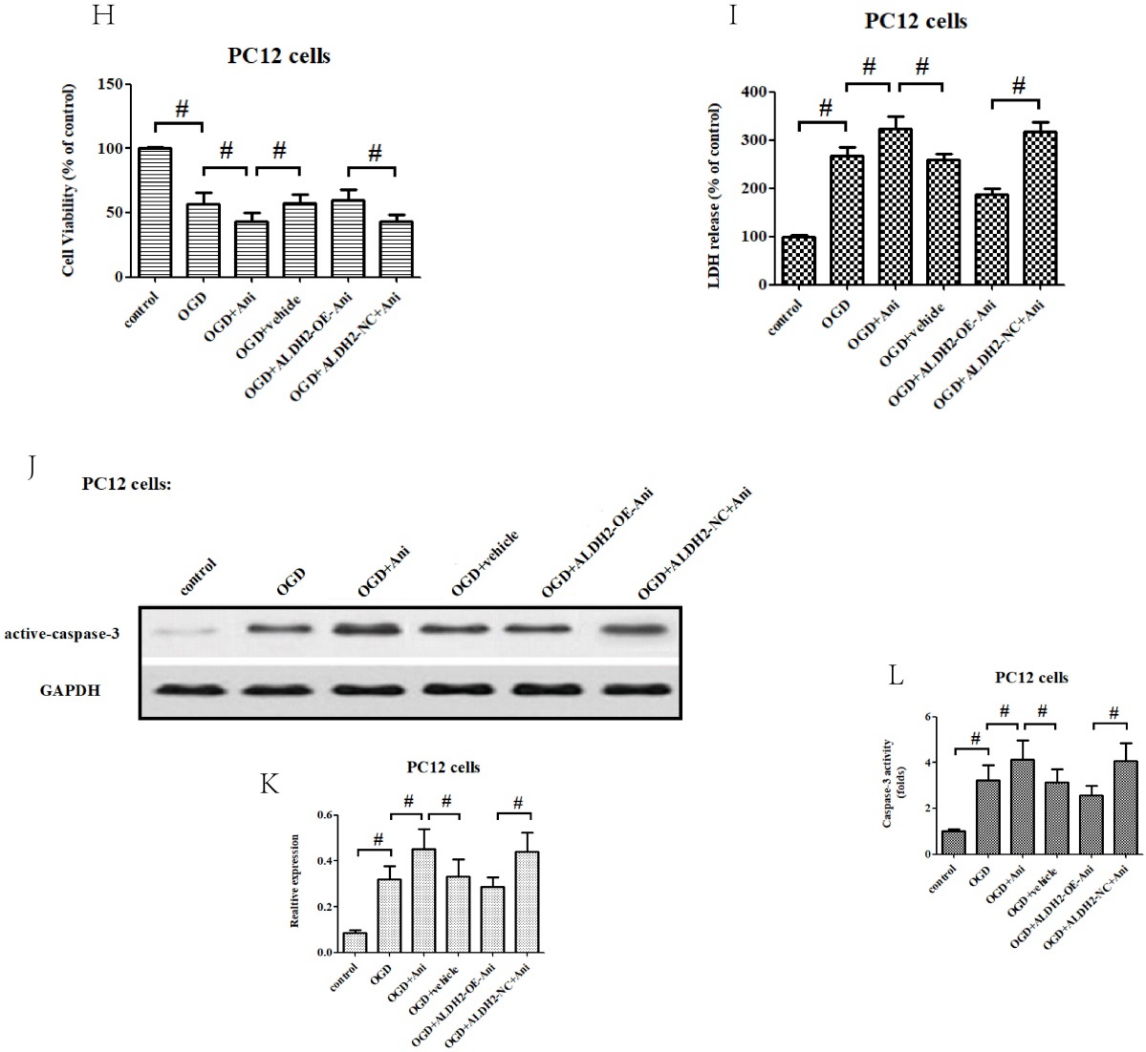
** ALDH2 conferred neuroprotection *via* suppressing JNK-mediated caspase-3 activation. A and B.** The effect of overexpression or knockdown of ALDH2 on phosphorylated JNK (p-JNK) in PC12 cells exposed to OGD damage. **C and D.** JNK specific inhibitor SP600125 (SP) promoted cell survival from ALDH2 deprivation under OGD conditions.** E.** SP significantly relieved caspase-3 activity measured by the ELISA kit. **F and G.** Western blots were used to analyze the effect of SP on activated caspase-3. **H and I.** JNK specific activator Anisomycin (Ani) increased cell death from ALDH2 reintroduction under OGD conditions. **J.** Ani significantly increased caspase-3 activity measured by ELISA kit.** K and L.** Selected graphs of active caspase-3 affected by Ani. Mean **±** SD was used to describe the data. ^#^ indicated *P < 0.05.*

As shown in** Fig. 7A and B**, p-JNK is correlated with OGD/R injury and inversely related to ALDH2. In addition, inhibition of JNK through SP600125 (SP) not only promoted cellular survival and alleviated LDH leakage (**Fig. 7C and D**), but also reversed the up-regulation of caspase-3 activity and expression under OGD/R stimulation. Moreover, SP rescued cellular viability from ALDH2 deprivation under OGD/R conditions (**Fig. 7E-G**). In comparison, cellular death and caspase-3 activity were all re-elevated after reactivating JNK in ALDH2-overexpressed PC12 cells with Anisomycin (Ani) (**Fig. 7H-L**).

**Figure 8:**
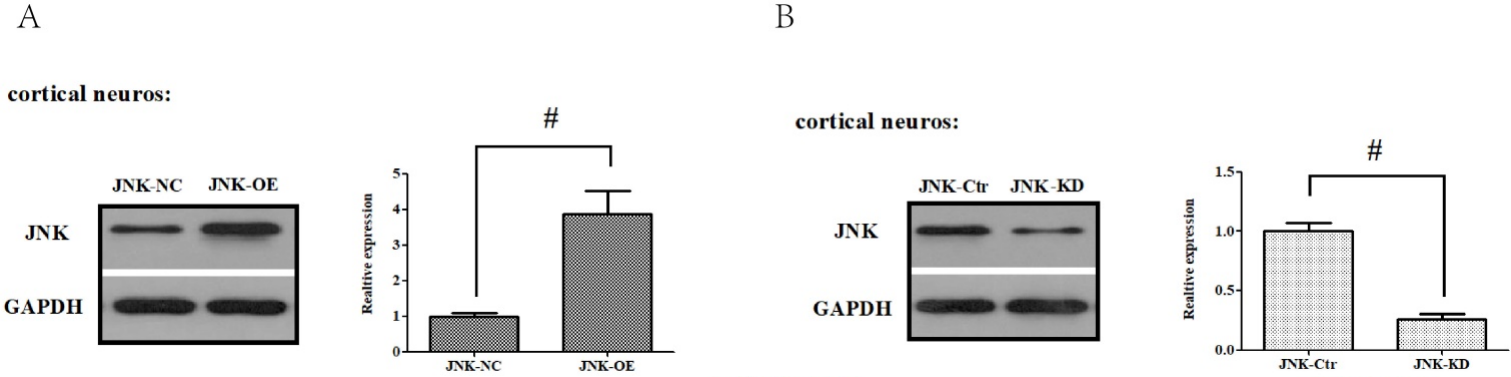

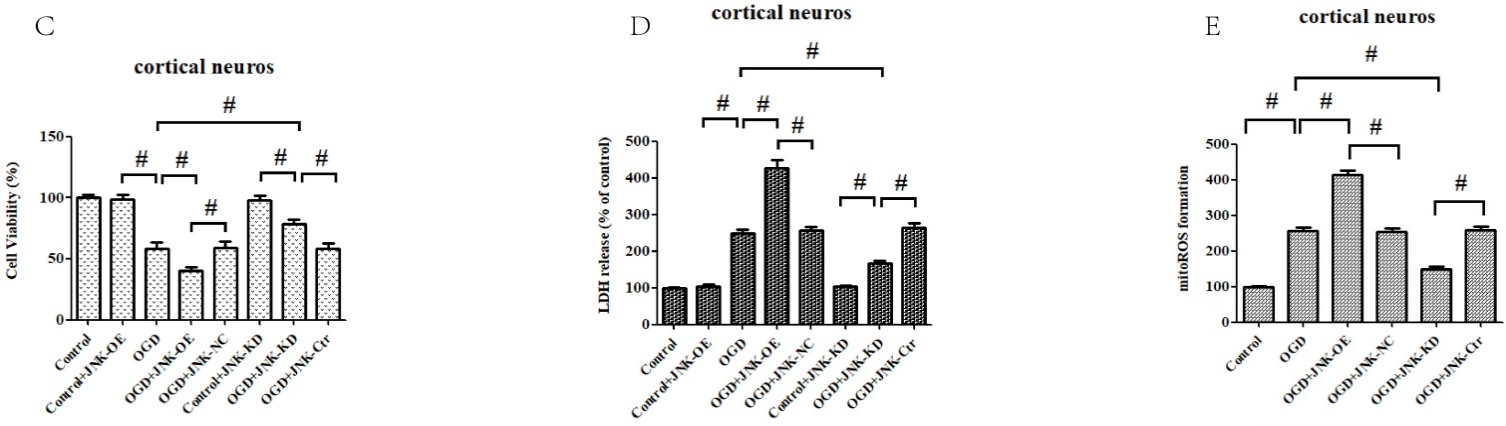

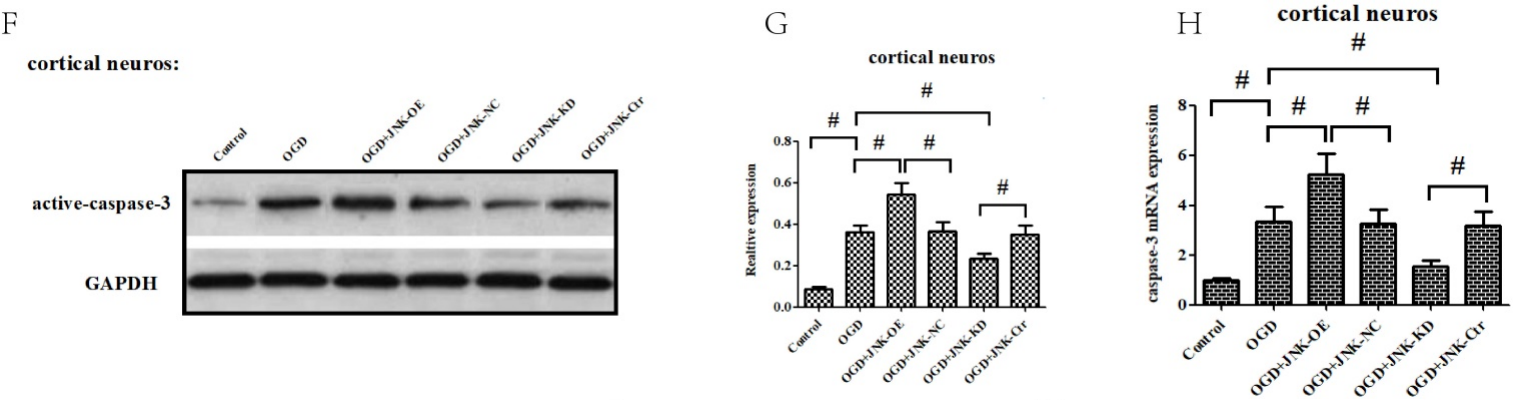

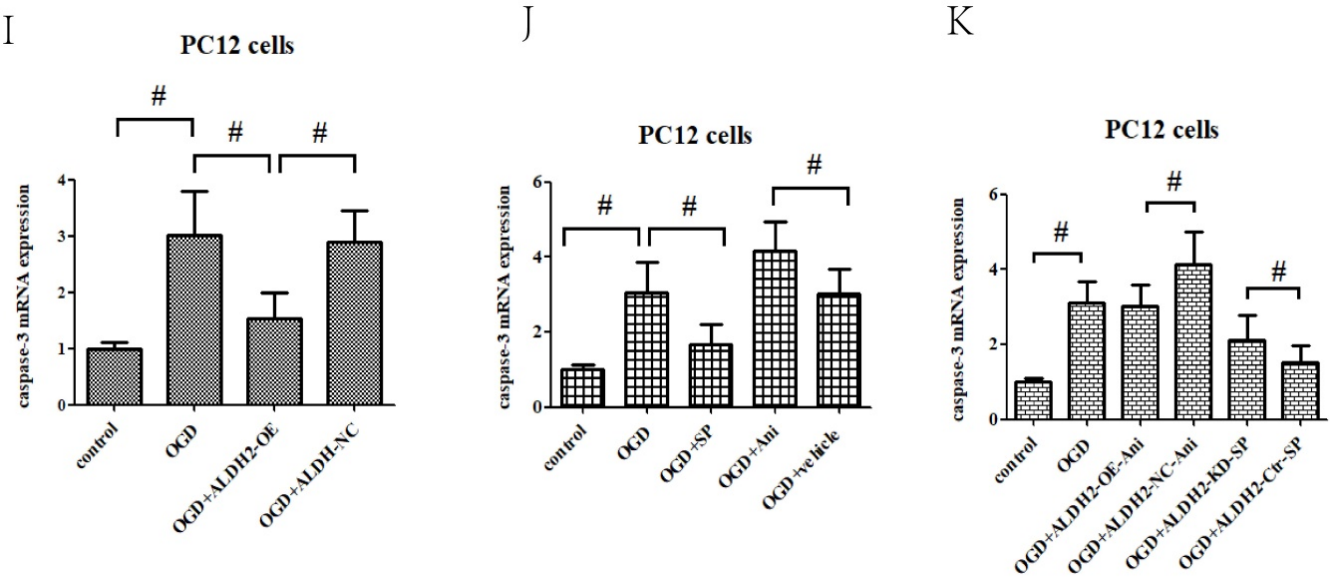

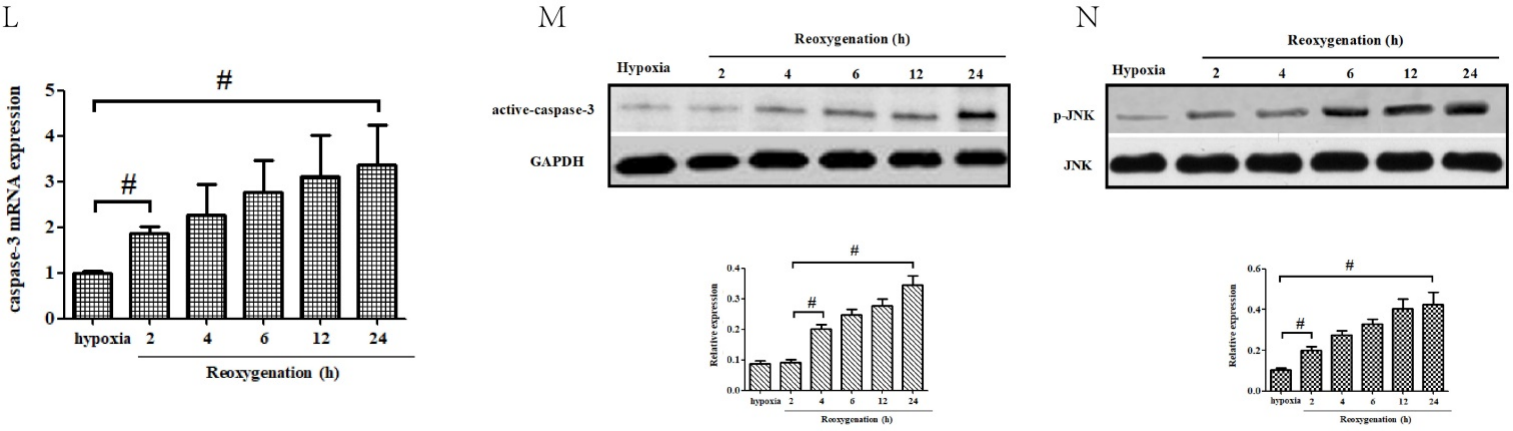

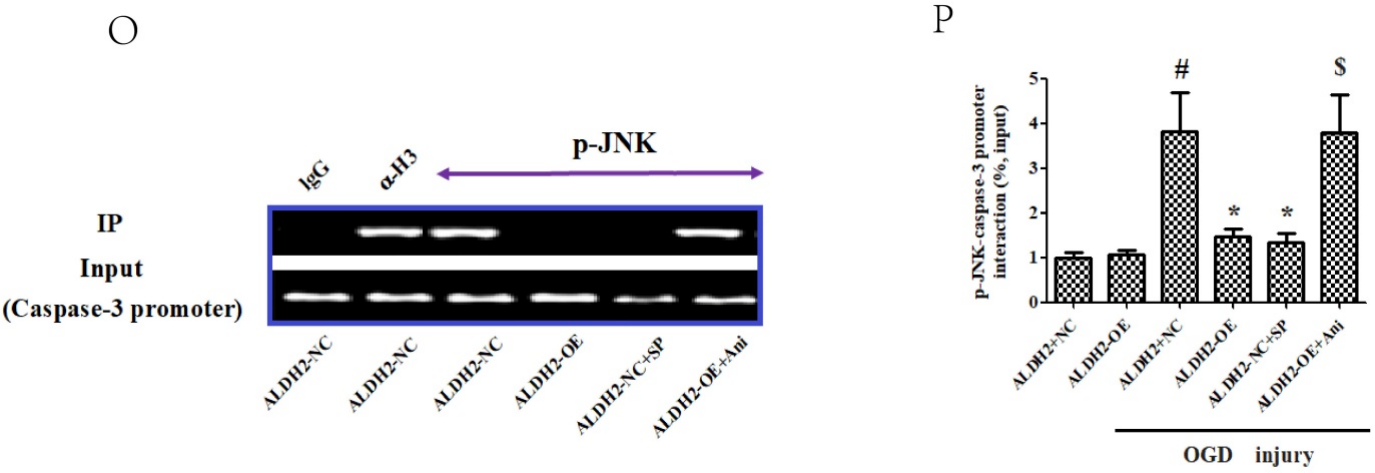
** ALDH2/JNK modified caspase-3 transcription. A and B.** Plasmid transfection or siRNA overexpressed or knockdown JNK expression, respectively, and representative gel blots of expression of JNK, using specific antibodies in the primary cortical neurons. **C and D.** Overexpression or inhibition of JNK modulated neuronal viability. **E**. Overexpression or inhibition of JNK regulated mROS generation. **F.** Overexpression or inhibition of JNK mediated caspase-3 activity. **G.** Representative gel blots of expression of active caspase-3. **H.** Overexpression or inhibition of JNK mediated the contents of caspase-3mRNA. **I-K.** qPCR assay for the differences of caspase-3 mRNA expression. **L.** The level of caspase-3 mRNA during hypoxia and at different time points after reoxygenation. **M and N.** Representative gel blots of expression and quantification analysis of phosphorylated JNK and active caspase-3. **O and P**, ChIP assay suggested interaction between the caspase-3 promoter and p-JNK under OGD injury. IgG was applied as a negative control, while anti-histone-3 (***α***-H3) was used as a positive control. Input corresponds to equal amplification of DNA in non-immunoprecipitated samples; IP demonstrated the amplified DNA band in the immunoprecipitated samples. Mean **±** SD was used to describe the data; ^*^P < 0.05, compared to OGD+ALDH2-NC group;^ #^*P <0.05,* compared to the ALDH2-NC group; ^$^ *P < 0.05,* compared to OGD+ALDH2-OE group.

To confirm these results, *in vitro* gain- and loss-of-function analysis of JNK were also performed to evaluate its effects on OGD/R exposure and relevant signaling pathways in the primary cortical neurons. Firstly, we established JNK knockdown and overexpression in the primary cortical neurons. As shown in **Fig. 8A and B**, JNK knockdown and overexpression cell lines were separately validated from protein levels compared with the vehicle control. Consumption of JNK then rendered improved cellular viability in the primary cortical neurons, with significantly increased survival rates and reduced LDH leakages as well as mROS formation compared with the vehicle control. Nevertheless, overexpression of JNK contributed to cellular damage (**Fig. 8C-E**). In addition, JNK overexpression or knockdown notably exacerbated or attenuated caspase-3 activation and transcription (**Fig. 8F-H**). Hence, these data fully indicated that JNK played a positive role in facilitating OGD/R -induced neuronal injury.

Since caspase-3 served as an apoptotic final executive molecule, we further determined how ALDH2/JNK modified the caspase-3 expression. At the beginning, caspase-3 gene transcription was measured, and it was found to have increased under OGD/R conditions. However, suppression of JNK or overexpression of ALDH2 significantly decreased the caspase-3 transcription. In the event of activated JNK in PC12 cells with ALDH2-overexpression, the level of caspase-3 transcription became re-elevated (**Fig. 8I-K**).

Moreover, our data showed that both p-JNK and caspase-3 mRNA increased at 2-h after reoxygenation while active caspase-3 elevated at 4-h after reoxygenation. This suggested that p-JNK and caspase-3 transcriptional activation preceded caspase-3 protein up-regulation (**Fig.8L-N**).

Finally, with the help of Chromatin Immunoprecipitation(ChIP) assay, our study indicated that p-JNK was bound to the caspase-3 promoter. Upon OGD/R stimulation, the two interacted with each other. However, a specific JNK inhibitor SP significantly inhibited this tendency which is much like the findings gained from ALDH2-overexpressed PC12 cells. In contrast, Ani (JNK activator) recreated the interaction between caspase-3 promoter and p-JNK in PC12 cells with ALDH2-overexpression (**Fig. 8O and P**).

## Discussion

Whether it is a cerebral surgery or not, anesthesiologists often face potential acute cerebral ischemia and hypoxia injury (perioperative stroke) which is a complex pathophysiological process. Although researchers have explored the pathological mechanisms and treatments for perioperative stroke injury in past decades, it is still a critical disease that threatens life and presents difficulties in medical scientific research.

Although the brain represents only 2% of the human body masses, it spends approximately 20% of oxygen and 25% of blood glucose to produce ATP 39. Thus, the brain has a particularly high density of mitochondria and is more susceptible to ischemic injury than other organs. Moderate formation of reactive oxygen species (ROS), evidenced in byproducts of oxidative phosphorylation, plays a pivotal role in maintaining the normal physiological function of neurons. However, excessive ROS generation from damaged mitochondria can trigger α, β-unsaturated carbonyls 40. Moreover, α, β-unsaturated aldehydes are derived from lipid peroxidation of biological membrane attributed to aldehyde buildup that has been related to the pathological mechanism of cerebral ischemic and neurodegenerative diseases 41, 42. Aldehydes-caused neurotoxicity has been implicated in cerebral ischemic injury. Numerous studies have demonstrated that excessive ROS generated by cerebral ischemic injury can cause accumulation of reactive aldehydes such as 4-hydroxynonenal (4-HNE), and malondialdehyde (MDA). The latter directly attacks cellular proteins and results in dysfunctions of proteins, which accounts for neuronal death 8, 9, 41. Mitochondrial aldehyde dehydrogenase 2 (ALDH2), a chief aldehyde catalyzing enzyme, clears the brain from deleterious reactive aldehyde load 8, 9, 41. ALDH2 serves as a core enzyme not only in converting toxic acetaldehyde to acetic acid but also clearance of other biogenic aldehydes such as 4-HNE in the brain 13, 14. Most importantly, ALDH2 is widely expressed in glial cells and neurons of the brain 10-12 while Alda-1 (N-(1,3-benzodioxol-5-ylmethyl)-2,6-dichlorobenzamid), a well-characterized ALDH2 agonist, have also been shown to provide efficacious neuroprotective effects against ischemic injury in the brain 41. Furthermore, ALDH2*2 allele frequencies were found to be significantly lower among stroke patients in several epidemiological analyses 43, 44. About half of East Asia's populations are heterozygous for ALDH2, which represents a higher risk factor for stroke 45. All the above findings suggest that ALDH2 has an important influence on the pathogenesis of brain ischemic injury, particularly in Asian individuals. Yet, the function of ALDH2 in brain stroke is still not fully understood because of limited research. As in other studies, we also found that ALDH2 expression experienced no alternation during the ischemic stage and was significantly down-regulated at different time points after reperfusion (the lowest level at 24h after reperfusion) in *in vivo* MCAO models. Furthermore, overexpression of ALDH2 not only significantly reduced infarct area and improved the post-ischemic neurological outcomes, but also notably decreased TUNEL-positive neurons in ischemic penumbra. This suggested that ALDH2 provided a robust neuroprotection against neuronal apoptosis.

Apoptosis, a strictly regulated path of programmed cell death occurs in neurological diseases including ischemic stroke 46. As we all know, acute ischemic stroke led to production of redundant ROS which resulted in damaged mitochondria inducing apoptotic pathways and neuronal death. Mitochondria-derived ROS (mROS) results in the opening of mitochondrial permeability transition pores (MPTP) and decreased levels of mitochondrial membrane potential (MMP). This eventually disrupts the structure and function of mitochondria 47, 48. In this study, it was demonstrated that cerebral ischemic reperfusion injury resulted in significant increase production of mROS whereas ALDH2 overexpression significantly inhibited mROS generation and was accompanied by preserving MMP and reducing MPTP opening. This implied a relationship between ALDH2 activity and ROS reduction. Additionally, it is still unclear if ALDH2 was downregulated during the reperfusion. As we all known, ROS has neuroprotective effect at low concentrations and is cytotoxic in high concentration. Previous studies have indicated that autophagy, a process for turnover of intracellular organelles and molecules, can induce ALDH2 degradation 49. Like a double-edged sword, mild autophagy is neuroprotective, whereas excessive autophagy is vital and neurotoxic. In fact, the same relationship also exists between surplus ROS production and autophagy. Moreover, our finding in this present study have shown that ROS levels increased almost by 3-fold after ischemia and OGD/R injury. Therefore, we can determine that the increase of ROS formation led to excessive autophagy and caused ALDH2 degradation. Furthermore, MPTP opening leads to Cyt C leakage and subsequent formation of activated caspase-9. It cleaves procaspase-3 to its active form, and finally leads to neuronal apoptosis. Based on the above background, inhibition of mitochondria-associated apoptosis might act as a therapeutic strategy for brain ischemic diseases. To gain more solid evidence, *in vivo* MCAO models were employed and it was found that overexpression of ALDH2 significantly suppressed mitochondria-related apoptosis. This was evidenced by the inhibition of caspase-9 and -3 activation, Cyt C release, and the increase of Bcl-2/Bax ratio. Meanwhile, ALDH2 reintroduction also significantly improved cellular viability and reduced LDH leakage as well as mitochondria-associated apoptosis *via* lowering mROS production both in the primary cortical neurons and PC12 cells in *in vitro* OGD/R models. Furthermore, genetic deprivation of ALDH2 had an opposite effect. Additionally, 4-HNE and MDA, generated during the process of oxidative stress, have all been discovered as classic toxic aldehydes. Their accumulation in the brain can induce neuronal apoptosis 41, 50. It was demonstrated in both *in vivo* MCAO models or *in vitro* OGD/R models that ALDH2 reintroduction or ALDH2 knockdown significantly reduced or aggravated intracellular accumulation of 4-HNE and MDA. Consequently, the anti-apoptotic effect of ALDH2 can be understood from the aspect of mROS, 4-HNE and MDA clearance. However, the critical molecular mechanisms of ALDH2 in the mitochondria-related apoptosis of neurons in ischemic stroke has not been clarified yet.

Several studies described that 4-HNE was regulated by c-Jun N-terminal protein kinase (JNK) indirectly, which functioned like signaling molecules, or directly by the kinase's active domains. JNK modulated the rate of cells and mediated neuronal injury in different ischemic models 51, 52. Since 4-HNE is a substrate of ALDH2, it is reasonable to hypothesize that ALDH2 mediates JNK activation. In this study, ALDH2 overexpression significantly alleviated MCAO-induced phosphorylation of JNK (activated JNK). However, the immunofluorescence technique showed that ALDH2 affected p-JNK expression mostly in neurons but not in astrocytes. To further strengthen this finding, we used primary cortical neurons and astrocytes to mimic the ischemic reperfusion injury in OGD/R model. After 6-h hypoxia and 24 h re-oxygenation, OGD/R significantly reduced their cellular viability. The gain- and loss-of function were subsequently carried out and we found that regaining of ALDH2 alleviated OGD/R-induced neurotoxicity while inhibiting ALDH2 had an opposite effect mainly in the primary cortical neurons. As expected, neither overexpression nor knockdown of ALDH2 had any influence on the cellular survival of cultured astrocytes. The reason for this unexpected result can be explained, as high levels of ALDH2 are primarily found in glial cells and neurons. In addition, we detected p-JNK only at 24 h after reperfusion. Astrocytes-mediated inflammation appeared 2-3 days after reperfusion, which was beyond our detection timeframe. Interestingly, OGD/R exposure triggered significant JNK activation both in the primary cortical neurons and PC12 cells. Meanwhile, genetic deprivation or pharmacological inhibitor of JNK *via* SP600125 (SP) not only promoted cellular survival and alleviated LDH release and mitochondria-related apoptosis, but also reversed the up-regulation of caspase-3 activity and expression upon OGD/R stimulation. Moreover, SP rescued cellular viability from ALDH2 deprivation under OGD/R conditions. In contrast, cellular apoptotic death and caspase-3 activity and expression were all re-elevated when JNK was reactivated in PC12 cells with ALDH2-overexpression by using a JNK specific activator, Anisomycin (Ani). These data indicated that ALDH2 conferred effective neuroprotection through inhibition of JNK phosphorylation and caspase-3 activation following OGD/R exposure.

There are three major molecular pathways which initiate apoptosis: intrinsic, extrinsic and the granzyme B pathway. All pathways lead to the activation of caspase-3 and the execution pathway that brings about the degradation of apoptotic cell debris 53. Thus, caspase-3 is well-characterized as the final executioner of the apoptotic pathway. Ample evidence has confirmed that JNK was responsible for the activation of caspase-3 activation 24, 25, 54. To investigate how JNK activate caspase-3, we looked into the possibility of whether JNK interacted with active caspase-3 in the ischemic penumbra of MCAO-treated rats. Similarly, ALDH2 overexpression significantly inhibited interaction between p-JNK and active caspase-3 induced by acute stroke. Moreover, it was found that both JNK antagonists and agonists not only affected the caspase-3 transcriptional activation, but also interfered with the effects of ALDH2 inhibition and expression on caspase-3 transcription. This gave us a hint that JNK is possibly involved in caspase-3 gene transcription. To further confirm this, we proved that p-JNK was bound to the caspase-3 promoter through Chromatin Immunoprecipitation (ChIP) assay. Additionally, our results also showed that phosphorylated JNK and caspase-3 transcriptional activation preceded caspase-3 protein up-regulation. Therefore, the data presented here identified that ALDH2 was the inhibitory system of JNK-required mitochondria-mediated apoptosis. Cerebral ischemia reperfusion injury significantly down-regulated the expression of ALDH2, resulting in the activated JNK pathway. Subsequently, activated JNK was translocated into the nuclei, promoting caspase-3 mRNA expression and leading to neuronal apoptosis.

**Figure 9:**
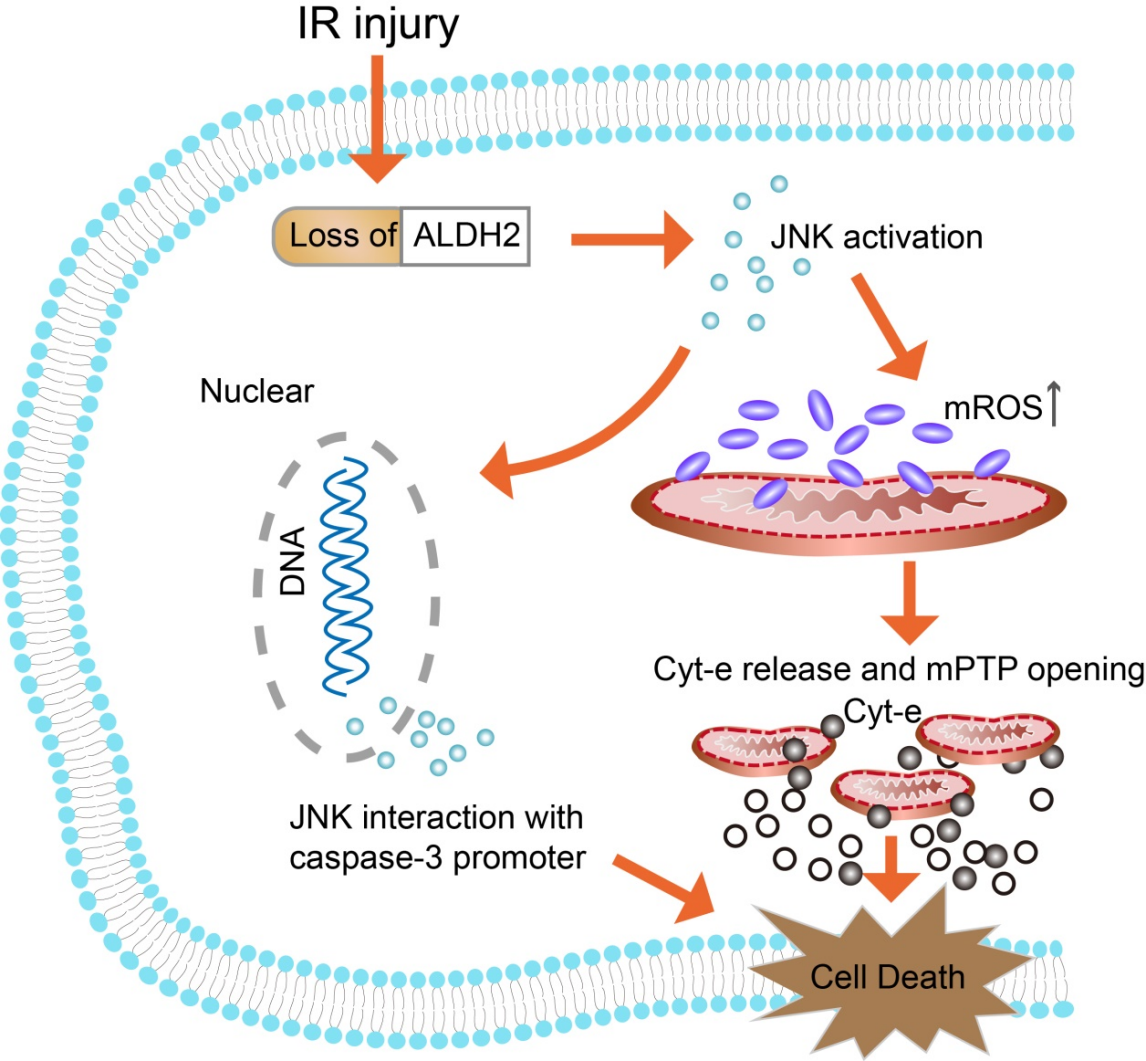
**Schematic representation of the proposed anti-apoptotic signaling pathways triggered by ALDH2 in ischemic neurons.** Cerebral ischemic reperfusion injury caused ALDH2 downregulation, leading to an increase in mitochondrial ROS (mROS) production. ROS served as a potential inducer of JNK. Then, activated JNK initiated the pro-apoptotic Cyt-C leakage from mitochondria into cytoplasm, launching the caspase-9 related mitochondrial apoptosis. Meanwhile, phosphorylated JNK was also bound to the caspase-3 promoter, leading to caspase-3 transcriptional activation. However, recovery of ALDH2 activity could cut off the excessive mROS and mitochondria-dependent apoptosis, providing prosurvival advantages for the brain in the context of ischemic injury.

In conclusion, it was indicated that down-regulation of ALDH2 following brain ischemic injury was associated with toxic reactive aldehydes accumulation. Then, it was proved that both overexpression and inhibition of ALDH2 regulated mitochondria-related apoptosis and that its underlying molecular mechanism involved the JNK/caspase-3 pathway (**Fig. 9**). Thus, ALDH2 likely affected the upstream of JNK-required mitochondrial apoptosis in the ischemic brain. Enhancing ALDH2 activity may become a novel target for patients with ischemic stroke.
